# Cost–utility analysis of risk-reducing strategies to prevent breast and ovarian cancer in BRCA-mutation carriers in Switzerland

**DOI:** 10.1007/s10198-021-01396-9

**Published:** 2021-11-12

**Authors:** Claudine Bommer, Judith Lupatsch, Nicole Bürki, Matthias Schwenkglenks

**Affiliations:** 1 University of Zurich, Zürich, Switzerland; 2 Department of Gynaecological Oncology, University Hospital Basel, Spitalstrasse 21, 4031 Basel, Switzerland; 3 Institute of Pharmaceutical Medicine (ECPM), University of Basel, Klingelbergstrasse 61, 4056 Basel, Switzerland; 4 Gynaecological Tumor Center, University Hospital Basel, Spitalstrasse 21, 4031 Basel, Switzerland

**Keywords:** *BRCA*, Cost-effectiveness, Risk reduction, Health economic modelling, Breast cancer, Ovarian cancer

## Abstract

**Objective:**

We aimed to identify the most cost-effective of all prophylactic measures available in Switzerland for women not yet affected by breast and ovarian cancer who tested positive for a *BRCA1/2 *mutation.

**Methods:**

Prophylactic bilateral mastectomy (PBM), salpingo-oophorectomy (PBSO), combined PBM&PBSO and chemoprevention (CP) initiated at age 40 years were compared with intensified surveillance (IS). A Markov model with a life-long time horizon was developed from the perspective of the Swiss healthcare system using mainly literature-derived data to evaluate costs, quality-adjusted life years (QALYs) and survival. Costs and QALYs were discounted by 3% per year. Robustness of the results was tested with deterministic and probabilistic sensitivity analyses.

**Results:**

All prophylactic measures were found to be cost-saving with an increase in QALYs and life years (LYs) compared to IS. PBM&PBSO were found to be most cost-effective and dominated all other strategies in women with a *BRCA1* or *BRCA2* mutation. Lifetime costs averaged to 141,293 EUR and 14.5 QALYs per woman with a *BRCA1 *mutation under IS, versus 76,639 EUR and 19.2 QALYs for PBM&PBSO. Corresponding results for IS per woman with a *BRCA2* mutation were 102,245 EUR and 15.5 QALYs, versus 60,770 EUR and 19.9 QALYs for PBM&PBSO. The results were found to be robust in sensitivity analysis; no change in the dominant strategy for either *BRCA*-mutation was observed.

**Conclusion:**

All more invasive strategies were found to increase life expectancy and quality of life of women with a *BRCA1* or *BRCA2 *mutation and were cost-saving for the Swiss healthcare system compared to IS.

**Supplementary Information:**

The online version contains supplementary material available at 10.1007/s10198-021-01396-9.

## Introduction

Breast cancer (BC) is the leading cause of years lost due to ill-health, disability or early death in women worldwide [1]. Ovarian cancer (OC) is associated with a disproportionately large number of deaths although OC is around one-tenth as common as BC. Healthcare costs associated with cancer treatment are growing rapidly, becoming an increasing burden for healthcare systems [2].

Germline mutations in the *BRCA1*- or *BRCA2*-gene are associated with high risk of OC and BC in affected women and characterised by early occurrence of BC (onset of disease typically after age 21 years) and OC (onset typically younger than 50 years). Women with a *BRCA1 *mutation have a cumulative risk of OC of 44% until age 80 years, of BC of 72% and of contralateral breast cancer (CBC) of up to 48%, depending on the age of primary BC onset [3, 4]. The cumulative risk for *BRCA2* to develop OC and BC by the age of 80 years is lower at 17% and 69% [3], as is the CBC risk (relative risk of 1.6 for *BRCA1* compared to *BRCA2*).

An inherited malignant tumour is found in 10–15% of epithelial OC [5]. High-grade serous carcinoma is the most common type of OC (including tubal and peritoneal cancer) in *BRCA*-mutation carriers [6], accounting for up to 70% of cases, and is also the most malignant form, causing most OC deaths. Its origin is often serous tubal carcinoma which spreads very early during the course of disease into the abdomen and presents as high-grade tumours with a resulting poor prognosis by the time they become symptomatic. There are few women (detected at an early FIGO stage) who survive for 10 years [7].

Between 5 and 10% of all BC cases are hereditary cancers in which a mutation in a *BRCA *gene is observed in up to 50% [8]. Mutation in either *BRCA1* or *BRCA2* not only predestines disease penetrance but also the molecular phenotype, treatment pathway and prognosis. About 70% of women with a *BRCA1 *mutation develop triple-negative BC (TNBC) [9, 10], i.e. they are estrogen-receptor (ER)-, progesterone-receptor (PR)- and human epidermal growth-factor-receptor-2 (Her2)-negative. In contrast, about 80% of women with a *BRCA2 *mutation develop hormone receptor-positive BC (HR + , i.e. ER + and/or PR +) [9, 10].

Women who tested positive for a *BRCA *mutation are confronted with the fear of developing cancer, which is emotionally, psychologically and socially challenging. They have to decide on different risk-reducing strategies. The least invasive option is intensive surveillance (IS; 6 monthly gynaecological consultations including breast imaging procedures). Alternatives are more radical invasive and irreversible surgical interventions. Prophylactic bilateral mastectomy (PBM) reduces BC risk by about 90% and prophylactic bilateral salpingo-oophorectomy (PBSO) OC risk by approximately 80% compared to IS [11]. Meta-analysis has shown a survival benefit for PBSO in women carrying a *BRCA*-mutation [12] but not for PBM [13]. So far, there is no effective screening method for OC [14]; transvaginal ultrasound combined with serum CA-125 test showed detrimental effects in one study [15] and promising results in another, implying a need for verification in larger cohort evaluations [16]. As an additional option, Tamoxifen, a selective estrogen receptor modulator, is approved in Switzerland as a chemoprevention agent for premenopausal women at high risk of BC. Tamoxifen is not expected to reduce the incidence of ER-negative BC and is therefore less recommended as chemoprevention for *BRCA1* mutation carriers than for *BRCA2* [17, 18].

Risk-reducing strategies to prevent BC and OC can be offered to women in Switzerland who underwent genetic counselling based on their family history and who were tested positive for a *BRCA*-mutation. The decision for a risk-reducing strategy is highly preference-sensitive and involves weighing the clinical effectiveness of interventions, including impact on survival and long-term quality of life, against adverse effects and other detrimental consequences. From a healthcare system perspective, understanding economic implications is of importance.

Cost–utility analysis in the United States [19] and Germany [20] has revealed that surgical prophylaxis is cost-effective for cancer-free *BRCA*-positive women at high risk of BC and OC. However, no such study is available for Switzerland. The situation in Switzerland may differ due to differences in the healthcare system, medical practice patterns and costs. To close this gap, our objective was to compare the costs and QALYs of different risk-reducing strategies available for healthy women with no personal history of BC or OC who have been tested positive for *BRCA1* or *BRCA2* in Switzerland and to estimate associated costs and health implications, using health-economic modelling.

## Methods

### Model

We adapted a decision-analytic Markov model from Muller et al. [20] in TreeAge Pro (TreeAge Software, LLC, Williamstown, MA, USA, version 2020 R1) for 40-years-old women with a confirmed *BRCA1* or *BRCA2* genetic mutation, but no history of BC or OC, and investigated the risk-reducing strategies available in Switzerland and recommended in current guidelines [21]: (1) IS (reference strategy, age-related imaging procedures and gynaecological consultations [22]), (2) PBM, (3) PBSO, (4) PBM&PBSO, and (5) chemoprevention (CP) to estimate cost-effectiveness expressed as cost per QALY gained and cost per life year (LY) gained. Age 40 years was chosen because by then most women have completed their family planning. Furthermore, current guidelines recommend PBSO at age of 35–40 years for pathogenic *BRCA1* and between 40 and 45 years for *BRCA2* variants [21]. The model was run separately for *BRCA1* and *BRCA2* to account for the different penetrance of the mutations and effectiveness of the risk-reducing strategies. *BRCA*-typical cancer phenotypes (HR /HER2 status and triple negativity) were considered in the clinical course and treatment. In the base case analysis, each strategy was analysed by itself, i.e. a counterfactual assumption of 100% uptake was made. Assumptions on mixed uptake were made in a scenario analysis.

The analysis was performed from the perspective of the Swiss healthcare system, i.e. all direct medical costs were considered irrespective of the payer. We used a hypothetical willingness-to-pay of CHF (Swiss Francs) 100,000 per QALY gained [23, 24]. A cycle length of one year and a duration of 60 cycles, implying a lifelong time horizon, were chosen to reflect the high survival rates of early diagnosed BC and the long-term clinical and monetary consequences of BC and OC. Costs and benefits were discounted by 3% per year in the base case analysis.

### Health states

**Fig. 1:**
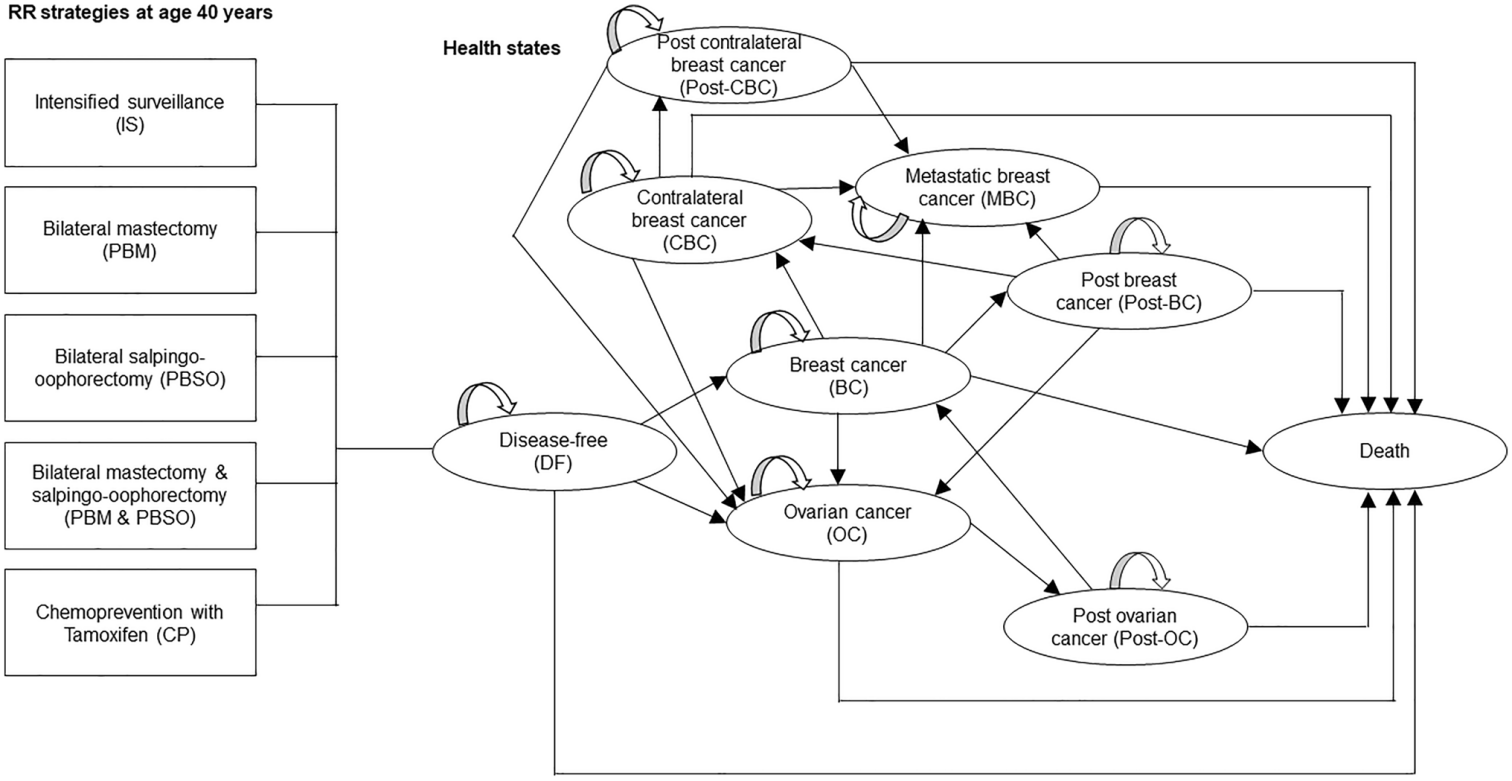
Overview of Markov model ( adapted from Muller et al. [20], arrows indicate possible transitions between health states)

The Markov model consisted of nine health states (Fig. 1): disease-free (DF), BC, CBC, metastatic breast cancer (MBC), post BC (Post-BC), post CBC (Post-CBC), OC, post OC (Post-OC), and death. Disease-free women either remained in the DF state, developed BC or OC and moved into the BC, respectively, OC state, or died due to other causes. Women with BC, who did not experience MBC, CBC, OC or died within the next 10 years, moved to the Post-BC state. Women in the Post-BC state continued in this state if they did not develop CBC, MBC, OC or die. Women who developed CBC and did not experience MBC, OC or death within 10 years after CBC diagnosis, moved to the Post-CBC state. Women remained in the Post-CBC state if they did not develop MBC, OC or die. IS mostly detects BC at an early stage (I–II) [10]. Therefore, MBC was assumed to be an event resulting from primary BC (no transitions from DF to MBC possible). Women who developed MBC stayed in this state until they died of the disease. Women with OC who did not die within 10 years after diagnosis moved to the Post-OC state. Few women develop metachronous BC after OC [25]. The prognosis of OC is generally poor and the median time of developing BC after OC was found to be approximately 9 years [25]. Hence, a transition to the BC state was only introduced in the Post-OC state. Women in the Post-OC state continued in this state if they did not develop BC or die.

### Model inputs

#### BRCA cohort-related input parameters (Supplementary Table S1)

For women undergoing PBM, we assumed that 5% of women choose an autologous breast reconstruction, while the remaining 0.95 prefer an implant-based breast reconstruction [26]. Furthermore, we assumed that an implant needs replacement every 10 years until the age of 70 years (according to warranty of implant manufacturers), and 50% of women have their breast appearance optimized by reshaping (about 3 months after the surgery).

For women undergoing BC surgery, the same assumptions were made as for PBM. Additionally, we assumed that 38% of women diagnosed with BC have a nodal involvement that requires radiotherapy (mean of *BRCA1* and *BRCA2*) [10], that the distributions of the molecular BC subtypes HR + , TNBC and Her2 + are 20%, 70% and 10% for *BRCA1* and 80%, 10%, 10% for *BRCA2* [9, 10], and that the BC case fatality in *BRCA*-mutation carriers is the same as for all BC patients.

In the context of PBSO, all premenopausal women were assumed to take hormone replacement therapy (estrogens plus progestine) up to the median age of natural menopause (51 years) to reduce postmenopausal symptoms and the increased risks of osteoporosis, cardiovascular disease (controversial results [27], 28) and possibly mental illness [29].

For ovarian cancer, we assumed that 63% of women have residual disease after OC surgery [30] and 73% of women relapse within 2 years [31].

#### Event probabilities (Table 1)

A literature search was performed in PubMed, available literature was reviewed and event probabilities derived from prospective, multi-centre/-national cohort studies were preferred over retrospective studies. Where available, data from Switzerland were used. Age-dependent yearly transition probabilities of developing BC, CBC, MBC, OC, dying from BC or OC, dying from any other cause, or remaining DF were estimated for each year of each risk-reducing strategy.

BC and MBC mortality rates were provided by the Swiss National Institute for Cancer Epidemiology and Registration (NICER). Data on OC mortality from Germany were used because, although Swiss data were comparable, they were only available for women aged 50–75 [32] and it is known that *BRCA*-mutation carriers have an earlier disease onset. Female, age-specific mortality in the general population were obtained from life tables published by the Swiss Federal Statistical Office [33]. Women were assigned cancer-specific probabilities of death up to 10 years after BC, CBC and OC diagnosis, then age-adjusted death probabilities were used in the post cancer states.

**Table 1:** Rates used to estimate transition probabilities for Markov model

		Rates (events of interest per person and year)			
		*BRCA1*^a^		*BRCA2*^a^	
Health States	Age	Base case	Sensitivity analysis (SE)^b^	Base case	Sensitivity analysis (SE)^b^
BC/CBC—> dead^c^	0–49	0.0209	0.002		
	50–69	0.0318	0.002		
	70 +	0.1066	0.005		
MBC—> dead^c^	0–49	0.2033	0.034		
	50–69	0.2558	0.048		
	70 +	0.2608	0.029		
OC—> dead [87]	15–44	0.0621	0.006		
	45–54	0.1086	0.007		
	55–64	0.1536	0.007		
	65–74	0.1983	0.008		
	≥ 75	0.2741	0.012		
DF—> BC [3]	21–30	0.0059	0.002	0.0048	0.002
	31–40	0.0248	0.003	0.0109	0.002
	41–50	0.0309	0.004	0.0330	0.005
	51–60	0.0269	0.007	0.0448	0.008
	≥ 60	0.0167	0.005	0.0247	0.005
DF—> OC [93]	30–39	0.0016	0.0007	0.0000	0.0000
	40–49	0.0155	0.0011	0.0012	0.0009
	50–59	0.0220	0.0013	0.0050	0.0014
	60–69	0.0165	0.0015	0.0135	0.0020
	≥ 70	0.0079	0.0011	0.0037	0.0013
BC—> CBC [3]	21–30	0.0119	0.007	0.0000	0.000
	31–40	0.0407	0.007	0.0186	0.007
	41–50	0.0209	0.005	0.0240	0.006
	51–60	0.0328	0.007	0.0224	0.006
	61–70	0.0117	0.007	0.0167	0.007
	71–80	0.0197	0.014	0.0132	0.012
BC/CBC—> MBC [94]		0.0278	0.002		
BC/CBC—> OC [95]		0.0185	Range: 0.012–0.058	0.0102	0.011
Post-OC—> BC/CBC [25]		0.0144	Range: 0.01–0.043	0.0095	Range: 0.007–0.0281
DF—> dead [33]	*Age-specific mortality probabilities of Swiss women*				

^a^If values for *BRCA2* were not stated separately in the table, then the same values as for *BRCA1* were used (reference publication collected *BRCA1/2* data, no differentiation between *BRCA1* and *BRCA2*)

^b^Standard Error (SE) based on 95% CI of the reference publication (95% CI higher limit – 95% CI lower limit approximated by 4*SE [96]), unless otherwise specified

^c^Data kindly provided by Swiss National Institute for Cancer Epidemiology and Registration (Nicer)

Abbreviations: DF (disease-free), BC (breast cancer), MBC (metastatic breast cancer), CBC (contralateral breast cancer), OC (ovarian cancer),—> (“to”)

#### Effectiveness of risk-reducing measures (Table 2)

A literature search was performed in PubMed, available literature was reviewed, and effectiveness data were preferably derived from prospective multi-centre/-national cohort studies for the surgical prophylactic interventions, given that randomized controlled trials (RCTs) are not feasible in this setting for ethical reasons. RCTs were used for efficacy data on chemoprevention. The ability of PBSO to reduce the risk of BC development is a controversial issue [34]. No prophylactic effect was found in the most recent data where PBSO was analysed as a time-dependent variable [34, 35]. We therefore decided for no prophylactic effect for BC for PBSO. Efficacy data of Tamoxifen for BC prevention were used from the randomized, controlled P-1 study [17] because meta-analysis [36, 37] included trials that allowed concomitant use of hormone replacement therapy, which is considered contraindicated in women under Tamoxifen treatment. No specific compliance adjustments were included in our model (daily Tamoxifen pill a 20 mg for 5 years) because effectiveness/efficacy data on screening and self-administrated drugs also include compliance effects if an intention-to-treat analysis is used which was the case in the P-1 study (0.24 of women discontinued the chemo-preventive drug prematurely). The effectiveness of Tamoxifen in specifically reducing HR + BC was used for primary BC. The proportion of HR+ BC in *BRCA1* increases with age [38], which may explain the chemo-preventive effect of Tamoxifen found in *BRCA1* for CBC [39]. *BRCA*-specific Tamoxifen efficacy values were used for CBC. It was assumed that the prophylactic effect of Tamoxifen is lifelong, as a risk-reducing effect was found up to 20 years [40].

**Table 2:** Risk reduction strategy input data

Risk reduction to develop		Base case ^a^	Sensitivity analysis ^b^ (SE)
OC	PBSO [95]	0.28^c^	0.14
	PBSO with prior BC [95]	0.14	0.14
BC	PBM [97]	0.09	0.09
	CP [17]	0.31^d^	0.06
CBC	CP—*BRCA1* [39]	0.44	0.15
	CP—*BRCA2* [39]	0.33	0.12

^a^Hazard ratio

^b^SE (standard error) based on 95% CI of the reference publication (95% CI higher limit – 95% CI lower limit corresponds to approximately 4*SE [96])

^c^Includes the remaining risk to develop peritoneal cancer

^d^Relative risk reduction to develop ER+ BC

OC (ovarian cancer), BC (breast cancer), CBC (contralateral breast cancer), PBSO (prophylactic bilateral salpingo-oophorectomy), PBM (prophylactic bilateral mastectomy), CP (chemoprevention)

#### Utility values (Table 3)

Quality-of-life adjustment was incorporated using the concept of utility with values ranging from 0 to 1 that express preference for a health state and are used to adjust a year of life for quality of life (zero representing death and one representing perfect health) [41]. Age-specific utility weights and utilities for each health state and risk-reducing strategy were obtained from published studies of general population samples.

**Table 3:** Utility input data

Description	Age	General population		Mutation carrier	p value ^f^	Method
		Base case	Sensitivity analysis ^e^	Mean values		
DF, age-specific [42]	30–39	0.901				EQ-5D
	40–49	0.871				
	50–59	0.842				
	60–69	0.823				
	70–79	0.790				
	≥ 80	0.736				
BC ^a^ [43]		0.637	± 20%			Meta-regression analysis
MBC ^b^ [43]		0.533	± 20%			Meta-regression analysis
OC ^c^ [99]		0.410	± 20%			Visual analogue scale
IS ^d^ [88]		0.960	± 5%	1.00	< 0.01	Time trade-off
PBM [88]		0.880	± 10%	0.88	< 0.11	Time trade-off
PBSO [88]		0.900	± 10%	0.95	< 0.01	Time trade-off
PBM & PBSO [19]		0.790	± 10%	0.84		Time trade-off
CP [88]		0.900	± 5%	0.95	< 0.01	Time trade-off

^a^Utility value of BC was derived using the constant early BC, mastectomy & reconstruction, chemotherapy non-specific, public community, EQ-5D (model 2)

^b^Utility value of MBC was derived using the constant MBC, chemotherapy non-specified, response non-specified, public community, EQ-5D (model 2)

^c^Utility value of OC: the following values were assumed over five years (a recurrence was assumed to occur within about 2 years after primary OC diagnosis [31] and overall survival after recurrence is about 30 months [100]) and averaged [99]: 0.56 (for the 1./2. year (newly diagnosed ovarian cancer—chemotherapy/grades 1–2 toxicity), 0.43 (for the 3./4. year (recurrent ovarian cancer—responding to chemotherapy/grades 1–2 toxicity); 0.08 (5. year (end stage)) resulting in an average calculated utility value of 0.41

^d^Intensified surveillance: utility value of annual MRI was used (annual mammography: 0.97)

^e^Estimated variation

^f^ Probability that there is no difference in the utility value between the general population and mutation carriers

Abbreviations: DF (disease-free), BC (breast cancer), MBC (metastatic breast cancer), OC (ovarian cancer), IS (intensified surveillance) PBM (prophylactic bilateral mastectomy), PBSO (prophylactic bilateral salpingo-oophorectomy), CP (chemoprevention)

Women were assigned a disease-related lower utility weight than in the DF state for the first 5 years after cancer diagnosis, after which they returned to an age-adjusted DF utility unless they fell ill again with cancer or died. Disease-related utility values were increased linearly within these 5 years after diagnosis. All health state utility values were age-adjusted using the multiplicative method, e.g. if a woman at age 50 developed BC, a utility weight of 0.842 (utility of a women aged 50 years [42])*0.637 (utility of BC [43]) was assigned in the first year she lived with BC [44]. We assumed the same utility weights for CBC as for BC.

Disutility weights of prophylactic surgical methods (PBM, PBSO, combination of both) were used for one year, while Tamoxifen was assumed to lead to a disutility for 5 years, after which age-adjusted general population utilities were used. One year of disutility was considered appropriate for prophylactic surgical interventions because the treated women are basically healthy women [45–48]. In contrast, cancer patients, after surgical removal of cancerous tissue (or procedures in case of complications or a later breast reconstruction), undergo further treatment(s), such as chemotherapy, radiotherapy, hormone therapy, psychological consultations. These treatments are physically and emotionally more stressful and for a longer period compared to a prophylactic surgery.

#### Healthcare costs (Supplementary Tables S2–S4)

Direct costs of medical care were estimated in EUR (exchange rate on 01 August 2019: 1 EUR = 1.10 CHF). Costs were categorized by inpatient (Swiss diagnosis-related group (DRG) system [49]) or outpatient setting (Tarmed national tariff system [50]) and listed medications reimbursed by the Swiss statutory health insurance [51]. Supplementary Tables S2–S4 detail the unit costs, procedures, follow-up visits, proportions and chemo-/targeted therapy used over a period of 10 years after BC/CBC and OC diagnosis, which were applied for cost estimation. Furthermore, based on the NICE Guideline 164 [22] which serves Swiss insurance companies as reference for reimbursement of services for women with a genetic mutation, IS was assumed to consist of semi-annual clinical consultations with a gynaecologist and age-related imaging procedures (30–59 years: annual mammography and MRI, 60–69 years: annual mammography, ≥ 70 years: mammography every two years). Women undergoing PBM were assumed to prefer an immediate breast reconstruction rather than later and participate in semi-annual clinical consultations. Women undergoing PBSO were expected to participate in IS. The same cost-related assumptions stated under PBM and PBSO were allocated to women undergoing PBM&PBSO, apart from one visit to a gynaecologist per year for early detection of other gynaecological diseases, follow-up of side effects of the prophylactic surgeries and surveillance of hormone replacement therapy. Women opting for CP were thought to consent to an annual vaginal ultrasound scan while taking Tamoxifen and participate in IS. The total cost estimate for each year of the 10 years after BC/CBC diagnosis was calculated by adding the chemo-/targeted therapy costs, under consideration of the *BRCA*-specific portions, with other costs relevant for that particular year (e.g. for the first year: cost of surgery, breast reconstruction, radiation, follow-up visits and procedures) (Supplementary Tables S3). Annual estimated costs of implant replacement were included following PBM and BC/CBC. Same costs were assumed for CBC as for primary BC. Standard chemo-/targeted therapy was used for the 1st and 2nd line for all cancers, further lines were modelled according to discussions with a local oncologist. Chemotherapy session costs were estimated based on the number of drugs administrated/session and the drug’s infusion period. Average annual chemo-/targeted therapy costs for MBC was estimated by dividing the total costs accumulated for each molecular subtype by its respective median overall survival (OS). A weighted average was calculated using the *BRCA*-specific distribution of subtypes. Cost allocation in the Post-BC and Post-CBC state depended on the risk-reducing strategy: semi-annual clinical consultations were assigned to PBM, cost of IS procedures for the PBSO, CP and IS cohort, 12-monthly clinical consultations for the combined PBM&PBSO strategy. A total cost estimate for each year of the 10 years following primary OC diagnosis was calculated by adding the corresponding chemo-/targeted therapy costs with other costs relevant for a particular year. Costs were adjusted from the 3rd to the 5th year to consider women suffering a recurrence of ovarian cancer (Supplementary Tables S4). Cost allocation in the Post-OC state depended on the risk-reducing strategy: annual clinical consultations were assigned to PBM and combined PBM&PBSO, cost of IS procedures for the PBSO, CP and IS cohort. A yearly vaginal ultrasound was allocated to each risk-reducing strategy for women in the Post-OC state. Women were assumed to adhere to the recommended consultations and procedures after prophylactic measures and development of cancer; adherence was not investigated during sensitivity analysis. Palliative care costs were included at transition from BC, MBC and OC to the death state**.**

### Model validation

A validation based on AdViSHE (Assessment of the Validation Status of Health-Economic decision models) was performed on the model before the final analyses were conducted to ensure confidence in the built model [52] (Supplementary Text 1).

### Sensitivity analyses

One-way and probabilistic sensitivity analyses were performed for all parameters subject to uncertainty to test the robustness of the results (Table 1, 2 and 3, Supplementary Tables S1, S2**)**. The uncertainty used for each parameter was established on literature-based standard errors, confidence intervals or ranges (minimum—maximum) or percentage variation was used. Probabilistic sensitivity analysis was run with 10,000 iterations, using gamma distributions for rates and costs, lognormal distributions for hazard ratios and beta distributions for utility values.

### Scenario analyses

In scenario analyses, variation in the proportion of women adopting different risk-reducing strategies was investigated. Furthermore, we examined the impact of the following alternative assumptions: initiation of risk reduction at 30 and 35 years instead of 40 years, immediate versus later breast reconstruction, duration of Tamoxifen’s prophylactic effect as short as only 5 years as not clearly known, duration of disutility up to 10 years for the surgical risk-reducing strategies (without linear increase) and OC mortality rate reduced by 30%.

## Results

### Base case

All risk-reducing strategies were found to be cost-saving for the Swiss healthcare system with an increase in QALYs and LYs compared to IS. PBM&PBSO was found to be most cost-effective and dominated all other strategies in women with a *BRCA1* or *BRCA2* mutation (Table 4). Lifetime costs averaged to 141,293 EUR and 14.5 QALYs per woman with a *BRCA1 *mutation under IS, versus 76,639 EUR and 19.2 QALYs for PBM&PBSO. Corresponding results for IS per woman with a *BRCA2 *mutation were 102,245 EUR and 15.5 QALYs, versus 60,770 EUR and 19.9 QALYs for PBM&PBSO. Supplementary Table S5 summarizes the total BC and OC cost estimations in the model.

**Table 4:** Cost-effectiveness base case results of different strategies for *BRCA1* and *BRCA2* mutation carriers for the prevention of breast and ovarian cancer

Strategy	Costs	QALYs	LYs	dCosts^a^	dQALYs^b^	dLYs^c^	ICER
*BRCA1*							
IS	141,293	14.48	18.62	Reference			abs. dominated
CP	136,957	15.24	18.89	− 4,336	0.76	0.27	abs. dominated
PBM	115,802	17.28	20.69	− 25,491	2.80	2.07	abs. dominated
PBSO	112,814	16.79	20.32	− 28,479	2.31	1.70	abs. dominated
PBM & PBSO	76,639	19.24	22.95	− 64,654	4.76	4.33	dominant
*BRCA2*							
IS	102,245	15.52	19.84	reference			abs. dominated
PBSO	97,091	16.85	20.41	− 5,154	1.33	0.57	abs. dominated
CP	78,478	17.58	21.44	− 23,767	2.07	1.60	abs. dominated
PBM	70,562	19.24	22.86	− 31,683	3.73	3.02	abs. dominated
PBM & PBSO	60,770	19.85	23.63	− 41,475	4.34	3.79	dominant

^a^dCosts: average difference in costs per women who opted for prophylactic measures compared to intensified surveillance (reference)

^b^dQALYs: average difference in QALYs per woman who opted for prophylactic measures compared to intensified surveillance (reference)

^c^dLYs: average difference in LYs per woman who opted for prophylactic measures compared to intensified surveillance (reference)

QALYs (quality-adjusted life years), LYs (life years), ICER (incremental cost-effectiveness ratio), abs. (absolutely), IS (intensified surveillance), PBM (prophylactic bilateral mastectomy), PBSO (prophylactic bilateral salpingo-oophorectomy), CP (chemoprevention)

### Sensitivity analyses

**Fig. 2:**
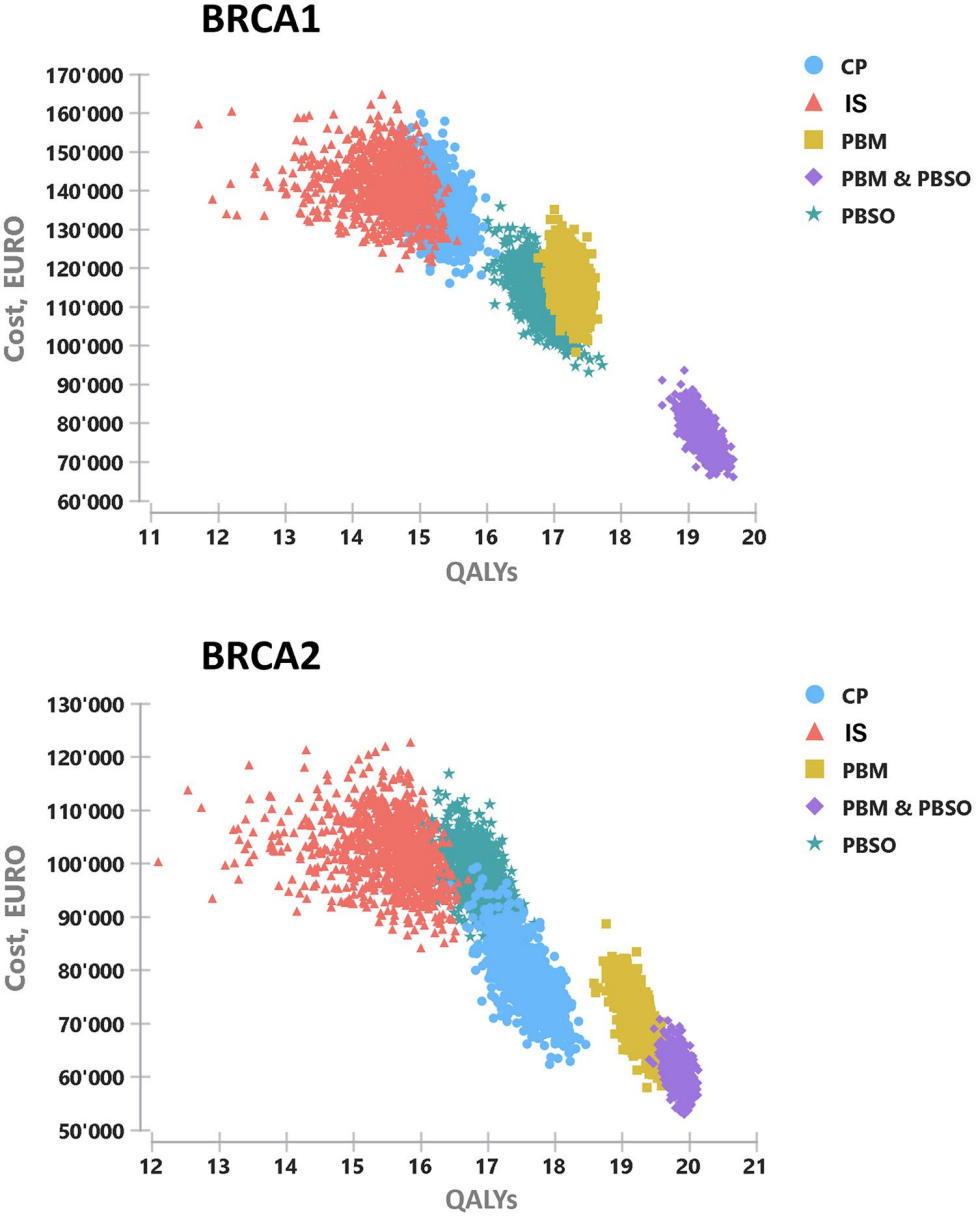
Cost-Effectiveness Scatterplot of the probabilistic sensitivity analysis for *BRCA1* (top) and *BRCA2* (bottom) with 10,000 iterations. CP (chemoprevention), IS (intensified surveillance), PBM (prophylactic bilateral mastectomy), PBSO (prophylactic bilateral salpingo-oophorectomy)

Tornado plots reveal that changes in incidence of OC after primary BC, costs of PBSO, HR of PBSO, costs of PBM with implant reconstruction, costs of implant replacement, utility values of surveillance and chemoprevention have most effects on the ICER (Supplementary Figures S1, S2). The base case results proved to be robust in the probabilistic sensitivity analysis and no change in the dominant strategy for either *BRCA*-mutation was observed (Fig. 2, Supplementary Figure S3).

### Scenario analyses

Decreasing absolute costs were observed if the risk-reducing strategies were initiated at ages 30 or 35 (Supplementary Table S6). A decrease in the OC mortality rate by 30% had little effect on costs and QALYs for *BRCA1* and *BRCA2* (Supplementary Table S7). Whether a breast reconstruction was performed immediately or later in women with BC had only minor effects on costs (Supplementary Table S8). An increase in the duration of disutility related to surgical risk-reducing strategies from 1 year (base case) to 10 years decreased the QALY gains achieved in comparison with IS (Table 5). The higher the proportion of women accepting surgical risk-reducing strategies, the greater the health benefits for the women and the more cost-savings for the Swiss healthcare system (Supplementary Figure S4).

**Table 5:** Results of sensitivity analysis of the duration of disutility related to surgical risk-reducing strategies

Strategy	Costs	QALYs	dCosts^a^	dQALYs^b^			ICER
	Duration of disutility for the surgical risk-reducing strategies						
	Base case (for 1 year)				for 5 years	for 10 years	
*BRCA1*							
IS	141,293	14.48	Reference				Abs. dominated
CP	136,957	15.24	− 4336	0.76	(for 5 years)		Abs. dominated
PBM	115,802	17.28	− 25,491	2.80	2.43	2.05	Abs. dominated
PBSO	112,814	16.79	− 28,479	2.31	2.01	1.73	Abs. dominated
PBM & PBSO	76,639	19.24	− 64,654	4.76	4.10	3.40	Dominant
*Brca2*							
IS	102,245	15.52	Reference				Abs. dominated
PBSO	97,091	16.85	− 5154	1.33	1.03	0.74	Abs. dominated
CP	78,478	17.58	− 23,767	2.07	(for 5 years)		Abs. dominated
PBM	70,562	19.24	− 31,683	3.73	3.34	2.93	Abs. dominated
PBM & PBSO	60,770	19.85	− 41,475	4.34	3.67	2.95	Dominant

^a^dCosts: average difference in costs per women who opted for prophylactic measures compared to intensified surveillance (reference)

^b^dQALYs: average difference in QALYs per woman who opted for prophylactic measures compared to intensified surveillance (reference)

QALYs (quality-adjusted life years), ICER (incremental cost-effectiveness ratio), abs. (absolutely), IS (intensified surveillance), PBM (prophylactic bilateral mastectomy), PBSO (prophylactic bilateral salpingo-oophorectomy), CP (chemoprevention)

## Discussion

Our model showed that risk-reducing surgery at the age of 40 years for women with BRCA1 or BRCA2 was more effective (increase life expectancy and quality of life) and cost-saving from the perspective of the Swiss healthcare care system compared to IS. The incidence of BC and OC was reduced by prophylactic interventions reducing the costs associated with cancer treatment. Due to the inability of IS to prevent BC and OC, the costs of repeated IS and cancer treatment exceeded the costs of any risk-reducing strategies and was therefore in disadvantage compared to the prophylactic measures. PBM&PBSO was found to be the most cost-effective strategy for women with a BRCA1 or BRCA2 mutation. It absolutely dominated all other risk-reducing strategies; this result was found to be robust in sensitivity analysis. Absolute costs were found to be higher for BRCA1 compared to BRCA2 for all strategies. This difference arises from the higher costs of BRCA1-associated BC treatment, which is mostly triple-negative, and the higher incidence of OC in BRCA1 than BRCA2 [3].

Several cost-effectiveness studies performed in other countries investigated risk-reducing strategies for women with a *BRCA1/2*-mutation and found that surgical interventions are beneficial to women’s health and induce cost-savings [20, 53–56]. Results of similar modelling studies in cancer-free *BRCA*-positive women were found to be quite heterogenous regarding estimated QALYs and LYs (Supplementary Table S9). However, they were consistent in the overall result that prophylactic surgical interventions were cost-effective. QALYs and LYs estimated in the present study fit the range of the other studies. Reasons for the heterogeneity between cost-effectiveness studies may include a) age of initiation of risk-reducing strategies, b) relative risk reduction of chemoprevention for *BRCA1* and *BRCA2* (differentiation by estrogen-receptor-status, *BRCA*-gene mutation), c) inclusion of adverse events for chemoprevention, d) risk reduction of oophorectomy to prevent BC, e) presence/absence of a transition from OC to BC, f) other modelling assumptions (duration of applied disutility), and g) *BRCA* cohort (separate analysis for *BRCA1* and *BRCA2* as in the present study or a combined approach which is not specific for a particular *BRCA* mutation). Our sensitivity analysis results regarding the duration of disutility induced by risk-reducing strategies indicated that the effect of alternative assumptions is highly influential and implications for conclusions derived from them need to be carefully evaluated for each cost–utility study.

Costs in the present study were found to be, depending on the *BRCA*-mutation, approximately 2–3 times higher than in a similar study performed in Germany where costs of standard chemotherapy were used in the estimation [20] and about half of the costs compared to the most recent study performed in the US [19]. US are known to have the most expensive healthcare system worldwide, followed by Switzerland [57]. A Swiss study found lower treatment costs for breast and ovarian cancer than our study. However, Wieser et al. [58] based their estimations on a different population. They investigated the Swiss female population while our study’s focus was on women with a *BRCA*-mutation who will cause *BRCA*-mutation-implied higher treatment costs.

An international study observed that on average about 50% of women at risk relied solely on IS, 49% underwent PBSO, 18% underwent PBM with considerable differences between countries [59]. No data were found about the acceptability of and preferences for the different risk-reducing strategies for women in Switzerland tested *BRCA*-mutation positive. The results of the CASCADE study involving German, French and Italian parts of Switzerland will clarify this subject [60]. The present study found that the higher the proportion of women who accept surgical risk-reducing strategies, the greater the health benefit for the women and cost-savings for the Swiss healthcare system. The most cost-effective strategy, PBM&PBSO, certainly is not acceptable for all women with a *BRCA*-mutation. A considerable proportion of women at risk rely solely on IS [59] and believe that IS allows an earlier diagnosis of cancer [61]. One study conducted in the Netherlands and one in Italy used surveys to investigate the effects of IS on quality of life in women with a *BRCA* mutation [61, 62]. Satisfaction with IS is generally high due to reduced concerns of cancer risk which resulted in a reported better quality of life [61]. Most of women who were found to have a false-positive result during IS that required further examination, understood the inconvenience and continued with scheduled examinations of IS [61]. Women experiencing psychological distress and increased anxiety due to IS opt for prophylactic surgery [61]. No difference in quality of life was observed between women at risk opting for IS or PBSO [62].

PBSO is suggested for all women with a *BRCA*-mutation as OC prognosis is bad and prophylactic surgery positively influences survival of women with a *BRCA *mutation (especially for *BRCA1*) [12, 63–65]. Results obtained in this study indicate that PBSO, an invasive intervention with little complications [66], is costs-saving in Switzerland. Related adverse effects, such as hot flashes and vaginal dryness, are associated with prematurely introduced menopause and symptoms are relieved by taking hormone replacement therapy until the age of natural menopause. Sexual habits and feelings after PBSO are impacted negatively [67] with high prevalence [68] though mitigated with hormone replacement therapy. Most women feel less distressed about developing OC after PBSO [61, 69].

Women contemplating PBM consider that today most women diagnosed early with BC do not die from it. A woman's self-image and body-image is more severely affected by PBM than by PBSO, which is usually not visible on the outside in contrast to PBM. A considerable proportion of women are dissatisfied with the breast appearance after PBM (scars) and feel embarrassed or less attractive when naked [59, 70, 71]. PBM was also associated with a high prevalence of discomfort in the breasts that impacted sexual sensations and enjoyment negatively [72, 73]. Dissatisfaction was also related with surgical complications [74] which occurred in up to 64% of patients [13, 75, 76], and unanticipated secondary surgeries after PBM [75]. Women are reluctant to undergo PBM despite its benefit [59]. However, women opting for PBM are generally satisfied with their choice because of reduced fear of developing BC [61, 77, 78].

Cancer causes physical (surgery, chemotherapy, side effects), mental (guilt towards offspring), emotional (frustration, sadness, fear) and social (disclosure stress, reaction of family members, conflicts, isolation, work-related concerns) distress for concerned women and their families affecting the life of everyone in the family, especially in emotional terms [79]. The decision whether and at what age a risk-reducing strategy is initiated requires rational decision-making based on individual preferences, copying style, anxiety level, family circumstances and cultural identity. It is a very personal and emotional decision about body parts that are currently still healthy and though they aren’t vital for survival, they define a woman's identity and may also femininity and personality [80]. Ultimately, the decision is based on weighing up the risk of developing cancer without any certainty of becoming ill in the future. Emotional and physical effects of undergoing/not undergoing risk-reducing intervention(s) with their associated consequences must be borne by affected women themselves, and therefore a woman can only decide on the significance and importance of the intervention(s) herself [80].

Strengths of the present analysis are that differences in penetrance, molecular subtypes of BC and efficacy of chemo-preventive risk reduction were considered by analysing the *BRCA1* and *BRCA2* population separately. Furthermore, care was taken during model development by early involvement of local clinical experts to reflect clinical practice [81]. Literature and adverse events of risk-reducing measures were carefully reviewed, procedures and chemo-/targeted therapy discussed with experts in the respective fields and costs were transparent and detailed collected.

The risk of endometrial cancer is increased in women ≥ 50 years of age while taking Tamoxifen [17] and ceases after the end of the intake [82]. Endometrial cancer was not considered as an adverse event of Tamoxifen since the treatment was assumed to start at the age of 40 years, with intake limited to 5 years. Deep vein thrombosis as an adverse event of Tamoxifen occurs during the active phase of intake [82] but with no proven attributable risk [17]. Cataract as a side effect of Tamoxifen was also not considered, as an excessive risk was only observed in older patients (> 65 years) [17]. Assuming that women who undergo PBSO for risk reduction only take hormone replacement therapy until their natural menopause, no increased risk from hormone replacement therapy (e.g. for BC) was expected and therefore also not included. Imperfect compliance of hormone therapy intake was not considered. Anaesthetic mortality is very rare and perioperative mortality in healthy women aged 40 years undergoing a prophylactic surgery is a rare event and therefore also not included [83]. Surgery-related complications related to mastectomies with subsequent breast reconstruction were observed in up to 64% of patients, independent of autologous or implant-based breast reconstruction [13, 75, 76], and considered as part of the optimization of the breast appearance. Laparoscopic PBSO in *BRCA*-mutation carriers was found to be safe with a low intra- and postoperative complication rate and therefore post-operative complications were not included [66].

Modelling studies generally are subject to limitations due to assumptions and uncertainties associated with input parameters. Assumptions made in the present study mainly concerned cost estimations such as proportions of women choosing an implant-based breast reconstruction. Furthermore, costs for diagnostic biopsies, side effects and complications of chemotherapy or costs in connection with secondary diagnoses/comorbidities, additional consultations to the standard oncologic and gynaecologic/clinical ones (psychologist/physiotherapist (lymph drainage), emergency or complications) were not considered, or only partially. Overall, costs may rather have been underestimated than overestimated.

IS was chosen as reference group as currently no data in Switzerland exists regarding risk-reducing strategy uptake. Mostly literature-based data from published prospective studies were used as the availability of Swiss data was very limited. Age-specific BC death rates of Switzerland were used in the present study regardless of the molecular phenotype or *BRCA*-status as corresponding Swiss data were missing. However, in international studies survival differences were observed depending on the molecular BC-type [84] and between *BRCA*-mutation carriers and *BRCA*-negative/sporadic BC patients, with generally a worse BC-specific survival for *BRCA*-mutation carriers [85]. An exception though is TNBC in women with a *BRCA *mutation which showed a better survival than the *BRCA*-negative counterpart. For Poly-ADP-Ribose-Polymerase-Inhibitor (PARPi), which was used in the cost estimation as it was recently approved in Switzerland as OC maintenance therapy for *BRCA*-mutation carriers, an estimate of median progression-free survival (PFS) was calculated of 52 months [86]. This is in discrepancy with the OC mortality rates derived from the general population with an estimated median OS of approximately 5 years that was used in the model [87]. This discrepancy’s impact was however addressed in the sensitivity analysis. In our model, only contralateral BC was considered as recurrent BC given a risk of up to 48% in BRCA mutation carriers which is very high compared to the risk of ipsilateral recurrence after mastectomy, radiation, if appropriate and systemic therapy [3]. Utility values derived from samples of the general population were chosen to make the results of the present study comparable to studies of other preventive programmes in the discussion of resource allocations. There is a controversial discussion about which utilities should be used, i.e. utilities derived from patients, their families, healthcare workers or the general population as differences were observed. Values derived from BRCA-mutation carriers are generally higher for the prophylactic measures [19, 88].

The reimbursement of prophylactic surgeries for *BRCA*-mutation carriers does often not cover the costs of executing hospitals in Germany [89]. There is a potential conflict of interest which may in the worst case impact the care and health benefit of *BRCA*-mutation carriers if this applies to Switzerland too. The present study indicates that from a Swiss healthcare perspective it had little effect on the costs whether a breast reconstruction was performed immediately within the primary BC surgery or in a separate later surgery. However, the time point of the breast reconstruction may result in a different outcome regarding cost coverage from a hospital point of view, which might be interesting to investigate for Switzerland. An immediate/single-stage breast reconstruction is certainly preferred from a woman’s perspective, if the woman’s medical condition and circumstances allow it, as it is associated with less surgeries and hospital stays than a two-stage reconstruction. An immediate breast reconstruction was found in a cost–utility analysis in the United States to be cost-effective from an insurance payer view [90]. An autologous breast reconstruction in Switzerland is more expensive than an implant-based. An implant-based breast reconstruction involves recurring costs due to repeated implant-replacements or complications such as fibrosis [91]. Women with an autologous breast reconstruction are more satisfied than women with an implant-based [92]. Therefore, it is of interest to investigate whether an implant-based breast reconstruction is cost-effective in the long term in comparison to an autologous breast reconstruction. This is even more true as cost-effectiveness analyses gain more and more importance in Switzerland for reimbursement decision-making and are encouraged and asked for by the Federal Office of Public Health, especially for non-drug healthcare services. A Federal Office section re-assessing currently reimbursed services has been established in 2017 and in the health technology assessments (HTAs) commissioned by this section, cost-effectiveness is explicitly considered as a key component. Cost-effectiveness analyses for Switzerland have been performed for decades in academic settings to provide guidance on value for money of medical services. Currently CHF 100,000 per QALY gained is the most frequently used tentative/hypothetical willingness-to-pay assumption in cost-effectiveness analyses for Switzerland based on several federal judgments in the past on expensive treatments [24]. Data about risk-reducing strategy uptake in Switzerland will be helpful to optimise the healthcare processes, survival, quality of life and ideally also the health-related costs of women with a *BRCA*-mutation in Switzerland.

### Conclusion

All risk-reducing strategies were found to increase life expectancy and quality of life of women with a *BRCA1* or *BRCA2* mutation and were cost-saving for the Swiss healthcare system compared to IS. PBM&PBSO was found to be the most cost-effective prophylactic strategy. PBSO is recommended considering the bad OC prognosis if the combined surgical strategy is not acceptable for a woman with a *BRCA*-mutation. PBM may be an option for women with a proactive copying style in addition to PBSO.

## Supplementary Information

Below is the link to the electronic supplementary material.

Supplementary file 1 (PDF 1710 KB)
