# Supplementary material for: Cost–utility analysis of risk-reducing strategies to prevent breast and ovarian cancer in BRCA-mutation carriers in Switzerland
Source: Eur J Health Econ. 2021 Nov 12;23(5):807–21. doi: 10.1007/s10198-021-01396-9 (PMC9170622; doi:10.1007/s10198-021-01396-9)
Supplement: Supplementary file 1 — (PDF 1710 KB) [file 10198_2021_1396_MOESM1_ESM.pdf]

SUPPLEMENT MATERIAL

Claudine Bommer[1,2], Judith Lupatsch[3], Nicole Bürki[4], Matthias Schwenkglenks[3]

Cost-utility analysis of risk-reducing strategies to prevent breast and ovarian cancer in *BRCA* mutation carriers in Switzerland

Affiliations of the authors
[1] University of Zurich, Zürich, Switzerland
[2] University Hospital Basel, Department of Gynaecological Oncology, Spitalstrasse 21, 4031 Basel, Switzerland
[3] University of Basel, Institute of Pharmaceutical Medicine (ECPM), Klingelbergstrasse 61, 4056 Basel, Switzerland
[4] University Hospital Basel, Gynaecological Tumor Center, Spitalstrasse 21, 4031 Basel, Switzerland

Corresponding author: Claudine Bommer, claudine.bommer@usb.ch

Index

Index **2**
Methods – Supplementary material **3**
Supplement Text 1: Model validation 3
Methods – Tables **3**
Supplement Table S1 Overview of cohort-specific variables and assumptions used in the model..... 3
Supplement Table S2 Input cost units of health care (EUR) 4
Supplement Table S3 Overview of breast cancer treatment for cost estimation & Health care costs (EUR) 6
Supplement Table S4 Overview of gynaecologic cancer treatment for cost estimation & Health care costs (EUR) 13
Results - Tables **15**
Supplement Table S5 Summary of total BC and OC cost estimations in the model 15
Supplement Table S6 Results of sensitivity analysis for *BRCA1* and *BRCA2* concerning age of prophylactic measures 16
Supplement Table S7 Results of sensitivity analysis for *BRCA1* and *BRCA2* concerning mortality rate of OC 17
Supplement Table S8 Results of sensitivity analysis for *BRCA1* and *BRCA2* concerning costs of a reconstruction at a later time point 18
Supplement Table S9 Overview of results (QALYs and LYs) that were found in modelling studies 19
Results - Figures **19**
Supplement Figure S1 Tornado diagrams showing parameters (excluding age) and their influence on the outcome of the cost-effectiveness for the different prophylactic strategies in comparison to the reference group in *BRCA1* mutation carriers 20
Supplement Figure S2 Tornado diagrams showing parameters (excluding age) and their influence on the outcome of the cost-effectiveness for the different prophylactic strategies in comparison to the reference group in *BRCA2* mutation carriers 21
Supplement Figure S3 Cost-effectiveness acceptability curve (CEAC) for *BRCA1* and *BRCA2*. 22
Supplement Figure S4 Results of scenario analysis showing different combinations of risk-reducing strategies for *BRCA1* and *BRCA2* 23
References **24**

Methods – Supplementary material

Supplement Text 1: Model validation

The following elements were discussed with or reviewed by respective experts of University Hospital Basel during conceptual validation: model (Dr. Nicole Bürki, Gynaecology & Genetic Counselling), OC-related surgical procedures (Dr. Homen Begovic), BC-related surgical procedures (Dr. Elisabeth Kappos, and Dr. Sabrina Gänsbacher), chemotherapy (Dr. Marcus Vetter) and radiation therapy (Dr. Markus Gross). The model's concept was cross-reviewed with other health-economic models [1, 2]. Literature was discussed (Dr. Nicole Bürki), input data/used formulas and the model in TreeAge were reviewed for correctness (Judith Lupatsch, PhD) and programming errors were solved before the analysis were properly executed. The outcome was verified by comparison with published literature [1, 3] and checked for plausibility, internal/technical validity/consistency (extreme values testing, meaningfulness of ranking order of the risk-reducing strategies).

Methods – Tables

Supplement Table S1 Overview of cohort-specific variables and assumptions used in the model

		Sensitivity analysis (SE) [a]	
Variables	Base case	Deterministic	Probabilistic (Distribution)
Proportion of women with IBBR	0.95 [4]	0.5-1.0	Beta: ±5%
Proportion of women undergoing a breast reshaping	0.5 [b]	0.4-0.6	Beta: ±10%
Proportion of women with lymph node positive BC (radiation therapy)	0.38 [5]	±20%	Beta: ±10%
Proportion of *BRCA1* carriers with an ER+ BC subtype	0.2 [5, 6]	0.18-0.28	Beta: ±10%
Proportion of *BRCA2* carriers with an ER+ BC subtype	0.8 [5, 6]	0.6-0.9	Beta: ±10%
Proportion of *BRCA* carriers with a Her2+ BC subtype	0.1 [5, 6]	±20%	Beta: ±10%
Proportion of women with residual disease after primary OC surgery	0.63 [7]	±10%	Beta: ±10%
Proportion of women having an OC recurrence	0.73 [8]	±10%	Beta: ±5%
Height (cm)	166 [c]	150-180	Normal: ±5%
Weight (kg)	60 [9]	50-80	Normal: ±15%
Proportion of women under chemotherapy in need of hematopoietic growth factor	0.5 [3]	±20%	Beta: ±10%

[a] Standard Error (SE), or range / estimated variation where otherwise specified, literature-based
[b] Clinical expert opinion
[c] https://www.conviva-plus.ch/?page=2046
Abbreviations: IBBR (implant-based breast reconstruction), BC (breast cancer), ER+ (estrogen-receptor positive), Her2+ (human epidermal growth factor receptor-2 positive), OC (ovarian cancer)

Supplement Table S2 Input cost units of health care (EUR)

	Base case	Sensitivity analysis [a]	Proportion	Source [b]
Surveillance & cancer follow-up				
Clinical consultation	127	±20%		Tarmed
Mammography	160	±20%		Tarmed
MRI Mamma/e	731	-20%/+50%		Tarmed
CT-Scan (thorax & abdomen)	616	±20%		Tarmed
Oncologic consultation (incl. blood sampling and laboratory analysis)	220	-20%/+50%		Tarmed
Mammography & Ultrasound	351	±20%		Tarmed
Vaginal ultrasound	120	±20%		Tarmed
Osteodensitometry (DEXA) [c]	84	±20%		Tarmed
Breast surgeries				
Prophylactic				
PBM with immediate ABR	18,648	-20%/+80%		DRG (J02B)
PBM with immediate IBBR	8,396	-20%/+60%		DRG (J24B)
Cancer				
Mastectomy (one-side) with immediate ABR *(base case)*	25,603	-20%/+80%		DRG (J01B)
Mastectomy (one/two-sided) with immediate IBBR *(base case)*	14,141	-20%/+60%		DRG (J06B)
Mastectomy (one-side) with ABR at a later time point *(only scenario analysis)*	28,623			DRG (J23Z, J02B)
Mastectomy (one-sided) with IBBR at a later time point *(only scenario analysis)*	16,099			DRG (J23Z, J24C)
Others				
Breast reshaping	6,373	-20%/+50%		DRG (J24B with cost reduction)
Replacement of implant (every 10 years)	9,837	-20%/+50%		DRG (J24A)
Radiation therapy				
Hypofractioned	10,592	±20%	0.7 [d]	Tarmed
Conventional fractioned	6,152	±20%	0.3	Tarmed
Gynaecological surgeries				
Prophylactic				
PBSO	7,842	-20%/+60%		DRG (N05C)
Cancer				
Salpingo-oophorectomy, hysterectomy, debulking in abdomen, pelvis, etc.	21,401	-20%/+50%		DRG (N01C)
Palliative care				
End of life treatment for metastatic BC / OC	34,147	±20%		DRG (A97D)
Chemotherapy associated costs				
Hospital costs / hour chemotherapy	105	±20%		Tarmed
Production tax / chemotherapy bag	53	±20%		
Material costs / chemotherapy medication	50	±20%		
Before each cycle: blood sampling, laboratory & choice of chemotherapy by oncologist	117	±20%		AL, Tarmed

Tolerability check 10 days after new chemotherapy regimen: blood sampling, laboratory & consultation with oncologist	218	±20%		AL, Tarmed
Chemotherapy-related medications [b]				
Antiemetics (every chemotherapy session)	71 per unit	±20%	1.0	SL
Hematopoietic growth factor (after each cycle)	1,533 per unit	±20%	0.5	SL
Bisphosphonates (for 5 years, every 6M)	275 per unit	±20%	1.0	SL
Paclitaxel premedication (excl. infusion period 90-180 min)	73 per session	±20%		SL

[a] Estimated variation
[b] DRG (code): https://www.swissdrg.org/de; using a base rate of EUR 9'236 (base case, Basel city) [10];
Tarmed: https://www.tarmed-browser.ch/de/leistungen; using a tax point value of EUR 0.83 (base case, Basel city) [11],
SL: http://www.spezialitätenliste.ch [12]
AL: https://www.bag.admin.ch/bag/de/home/versicherungen/krankenversicherung/krankenversicherung-leistungen-tarife/Analysenliste.html
[c] For HR+ BC with Anastrozole treatment: in the year of diagnosis and every two years during aromatase-inhibitor therapy
[d] Clinical expert opinion
Abbreviations: DRG (Diagnosis Related Groups), AL: "Analyseliste" (laboratory list), SL: "Spezialitätenliste" (Swiss medication list), MRI (magnetic resonance imaging), CT (computer-tomography), PBM (prophylactic bilateral mastectomy), PBSO (prophylactic bilateral salpingo-oophorectomy), BC (breast cancer), OC (ovarian cancer), M (months), SE (standard error), ABR (autologous breast reconstruction), IBBR (implant-based breast reconstruction)

Supplement Table S3 Overview of breast cancer treatment for cost estimation & Health care costs (EUR)

BREAST	Medication	Application	10 years										Proportion	Costs (EUR) [a]
**BC & CBC**			1	2	3	4	5	6	7	8	9	10		
**Surgery**			x										Autologous 0.05, implant 0.95 [4])	Supplement Table S2
**Radiation**			x										Lymph node positive: 0.38 [5]	Supplement Table S2
**Follow-up visits**			Every 3M clinical consultation, yearly mammography & ultrasound		Every 6M clinical consultation), yearly mammography & ultrasound			Every 12M clinical consultation, yearly mammography & ultrasound						Supplement Table S2
**Chemo- & targeted therapy** [d]														
HR+	Alkylans (e.g. cyclophosphamide, 500mg/m^2; 60min)	4 cycles (every 3 weeks)	x										*BRCA1*: 0.2 *BRCA2*: 0.8 [5, 6]	36 per 1000 mg unit
	Taxane (e.g. docetaxel, 75mg/m^2, 60min)	4 cycles (every 3 weeks)	x											735 per 180 mg unit
	Platin-bond (e.g. carboplatin, 6 AUC mg/ml*Min; 15-60min)	4 cycles (every 3 weeks)	x											126 per 600 mg unit
	Aromatase inhibitor (e.g. anastrozole)	1 mg daily for 5 years; lymph node positive for 10 years	x	x	x	x	x	(x)	(x)	(x)	(x)	(x)		238 per 98 pills of 1mg

BREAST	Medication	Application	10 years										Proportion	Costs (EUR) [a]
**BC & CBC** *(continued)*			1	2	3	4	5	6	7	8	9	10		
TNBC	Anthracycline (e.g. doxorubicin, 60mg/m^2; 10min)	4 cycles (every 3 weeks)	x										*BRCA1*: 0.7 *BRCA2*: 0.1 [5, 6]	141 per 100 mg unit
	Alkylans (e.g.cyclophosphamide, 600mg/m^2; 60min)	4 cycles (every 3 weeks)	x											36 per 1000 mg unit
	Abraxane (e.g. paclitaxel, 80mg/m^2; 60min)	4 cycles of one doses/week for 3 weeks (i.e. 12x totally), then one-week break	x											226 per 300 mg unit
	Platin-bond (e.g. carboplatin, 6 AUC mg/ml*Min; 15-60min)	4 cycles (every 3 weeks)	x											126 per 600 mg unit
Her2+	Taxane (e.g. docetaxel, 75mg/m^2, 60min)	6 cycles (every 3 weeks)	x										*BRCA1*: 0.1 *BRCA2*: 0.1 [5, 6]	735 per 180 mg unit
	Platin-bond (e.g. carboplatin, 6 AUC mg/ml*Min; 15-60min)	6 cycles (every 3 weeks)	x											126 per 600 mg unit
	Trastuzumab (e.g. Herceptin 6mg/kg, 30min)	for one year, every 3 weeks (i.e. 17x totally)	x											1,905 per 440 mg unit
	Pertuzumab (e.g. Perjeta 420mg, 30-60min)	for one year, every 3 weeks (i.e. 17x totally)	x											3,004 per 420 mg unit

BREAST	Medication	Application	10 years										Proportion	Costs (EUR) [a]
**MBC**			1	2	3	4	5	6	7	8	9	10		
**Diagnostics** (CT-Scan)			x											Supplement Table S2
**Follow-up visits**			Every 3M clinical consultation & CT-Scan											Supplement Table S2
**Chemo- & targeted therapy** [d]														
HR+ (median OS: ~60M [13], ~3 years of endocrine treatment + ~2 years systemic treatment for *BRCA* mutation carrier until palliative care according to oncologist)														
1st	Aromatase inhibitor (e.g. anastrozole)	until progression (PALOMA-1/TRIO-18 study; median PFS[b] 20.2M) [14]	x	x										238 per 98 pills of 1mg
	CDK4/6 inhibitor (palbociclib e.g. ibrance)		x	x										3,450 per 21 pills a 125mg (i.e. monthly supply)
2nd	Anti-Estrogen (e.g. fulvestrant)	until progression (PALOMA-3 study; median PFS 4.6M [15]); 1th months: 3x, then 1x/month			x									587 per application
3rd	Aromatase inhibitor (e.g. exemestane, 25mg, daily)	Until progression (BOLERO-2; median PFS 7.8M) [16]			x									264 per 90 pills of 25mg
	mTOR inhibitor/everolimus (e.g. afinitor, 10mg/daily)					x								3,689 per 30 pills of 10mg
4th	Taxane (e.g. docetaxel, $100mg/m^2$, 60min)	6 cycles (every 3 weeks)				x								735 per 180 mg unit
5th	Anthracycline (e.g. doxorubicin, $75mg/m^2$; 10min)	6 cycles (every 3 weeks)					x							141 per 100 mg unit

BREAST	Medication	Application	10 years										Proportion	Costs (EUR) [a]
**MBC**			1	2	3	4	5	6	7	8	9	10		
TNBC (median OS: ~22M [17, 18])														
PDL-1 negative													0.6 [18]	
1st	Anthracycline (e.g. doxorubicin, 75mg/m^2; 10min)	6 cycles (every 3 weeks)	x											141 per 100 mg unit
2nd	Taxane (e.g. docetaxel, 100mg/m^2; 60min)	6 cycles (every 3 weeks)	x											735 per 180 mg unit
3rd	PAPRi (e.g. olaparib, 600 mg/day)	until progression (OlympiAD study; median PFS 7.0M [19])		x										4,873 per 112 pills a 150 mg
4th	Platin-bond (e.g. carboplatin, 6 AUC mg/ml*Min; 15-60min)	6 cycles (every 3 weeks)		x										126 per 600 mg unit
PDL-1 positive													0.4 [18]	
1st	Abraxane (e.g. nab-paclitaxel, 100mg/m^2, 30min)	6 cycles, applied on days 1, 8, 15 of each 28-day cycle	x											374 per 100 mg unit
	Atezolimumab (anti-PDL-1, 840mg, 30min)	Applied on day 1 and 15 of each 28-day cycle, until progression (IMpassion130 study; median PFS 7.5M [18])	x											4,737 per 1200 mg unit

BREAST	Medication	Application	10 years										Proportion	Costs (EUR) [a]
**MBC** *(continued)*			1	2	3	4	5	6	7	8	9	10		
2nd	Anthracycline derivative (e.g. caelyx, 50mg/m^2; 60min)	6 cycles (every 4 weeks)	x											583 per 20 mg unit
3rd	PAPRi (e.g. olaparib, 600 mg/day)	until progression (OlympiAD study; median PFS 7.0M [19])		x										4,873 per 112 pills a 150 mg
4th	Platin-bond (e.g. carboplatin, 6 AUC mg/ml*Min; 15-60min)	6 cycles (every 3 weeks)		x										126 per 600 mg unit
Her2+ (median OS: ~54M; [20, 21]) [c]														
1st	Taxane (e.g. docetaxel, 100mg/m^2; 60min)	6 cycles, every 3 weeks	x											735 per 180 mg unit
	Trastuzumab (e.g. Herceptin 6mg/kg, 30min)	until progression, every 3 weeks (CLEOPATRA study; median PFS 18.5M [22])	x	(x)										1,905 per 440 mg unit
	Pertuzumab (e.g. Perjeta 420mg, 30-60min)		x	(x)										3,004 per 420 mg unit
2nd	Trastuzumab emtansine (e.g. Kadcyla, 3.6mg/kg, 60min)	until progression, every 3 weeks (EMILIA study; median PFS: 9.6M [23])		(x)	x									2,799 per 160 mg unit

BREAST	Medication	Application	10 years										Proportion	Costs (EUR) [a]
**MBC** *(continued)*			1	2	3	4	5	6	7	8	9	10		
3rd	Platin-bond (e.g. carboplatin, 6 AUC mg/ml*Min; 15-60min)	6 cycles, every 3 weeks			x									126 per 600 mg unit
	Trastuzumab (e.g. Herceptin 6mg/kg, 30min)	until progression, every 3 weeks (assumption by oncologist: 9M)			x	(x)								1,905 per 440 mg unit
4th	Anthracycline derivative (e.g. caelyx, 50mg/m^2; 60min)	6 cycles (every 4 weeks)				x								583 per 20 mg unit
	Trastuzumab (e.g. Herceptin 6mg/kg, 30min)	until progression, every 3 weeks (assumption by oncologist: 6M)				x								1,905 per 440 mg unit
5th	Capecitabinum (e.g. Xeloda, 2000mg/m^2 daily)	6 cycles (twice daily for 2 weeks, then one-week break in the 3 weeks cycle)				x								278 per 120 pills a 500mg
	Tyrosine kinase inhibitor (lapatinib e.g. Tyverb, 1250mg daily)	until progression (assumption by oncologist: 4M)				x								2,557 per 140 pills a 250mg

BREAST	Medication	Application	10 years										Proportion	Costs (EUR) [a]
**MBC** *(continued)*			1	2	3	4	5	6	7	8	9	10		
6th	Vinorelbine (e.g. navelbine, 30mg/m^2, 30min)	4-6 cycles (weekly)					x							149 per 50 mg unit
	Trastuzumab (e.g. Herceptin 6mg/kg, 30min)	until progression, every 3 weeks (assumption by oncologist: 4M)					x							1,905 per 440 mg unit

[a] Medication costs were obtained from the Swiss medication list, http://www.spezialitätenliste.ch [12], sensitivity analysis of medication costs: ±20% (estimated variation)
[b] PFS: progression-free survival
[c] Anti-Her2+ therapy beyond progression improves survival [24-27]
[d] Specifications for chemotherapy (cost) calculations: The amount of chemotherapy required for the cost estimate [mg] to reach a specific target [mg/m^2] was calculated using du Bois' formula for calculating body surface area [m^2] (body surface [m^2]=0.007184 x height $[cm]^{0,725}$ x weight $[kg]^{0.425}$) [28] and assuming that the average Swiss woman is 166cm tall (https://www.conviva-plus.ch/?page=2046) and of normal weight (60kg) [9] resulting in a body surface area of 1.67m^2.
To achieve a target AUC of 6 for carboplatin chemotherapy, the following assumptions were made: The mean age of breast and ovarian cancer diagnosis for women with a *BRCA* mutation is 46 respectively 57 years [29], with a normal creatinine level in the blood serum of 1.1 mg/dL resulting in a glomerular filtration rate (GFR) of 60.5mL/min or 53.4mL/min, respectively, using the formula GFR[mL/min] = (((140-age[years])*weight[kg]) / (72*serum-creatinine[mg/dL]))*0.85(for women). Using the Calvert-formula (i.e. carboplatin-dose [mg] = TargetAUC [mg/ml*min] * (GFR [mL/min] + 25)) [30] resulted in a carboplatin dosage of 513mg for breast respectively 471mg for ovarian cancer, which was used for the cost estimation.
Abbreviations: BC (breast cancer), CBC (contralateral breast cancer), M (months), HR+ (hormone receptor positive), TNBC (triple negative breast cancer), HER2+ (human epidermal growth factor receptor 2-positive), PDL-1 (programmed death ligand 1), CT (computer-tomography)

Supplement Table S4 Overview of gynaecologic cancer treatment for cost estimation & Health care costs (EUR)

GYNAECOLOGIC	Medication	Application	10-years tunnel										Proportion	Costs [a] (EUR)
**OC**			1	2	3	4	5	6	7	8	9	10		
**Diagnosis** (CT-Scan)			x											Supplement Table S2
**Surgery**			x											Supplement Table S2
**Follow-up visits**			Every 3M clinical consultation including vaginal ultrasound		Every 6M clinical consultation including vaginal ultrasound			Every 12M clinical consultation including vaginal ultrasound						Supplement Table S2
**Chemotherapy & targeted therapy** [b]	Platin-based (e.g. carboplatin, 6 AUC mg/ml*Min; 15-60min)	6 cycles, every 3 weeks	x											126 per 600 mg unit
	Taxane-based (e.g. paclitaxel, 175mg/m^2; 60min)	6 cycles, every 3 weeks	x											226 per 300 mg unit
	PAPRi (e.g. olaparib, 600 mg/day; SOLO-1 study)[31]	for 2 years	x	x										4,873 per 112 pills a 150 mg
	Anti-VEGF (bevacizumab, e.g. Avastin, 7.5mg/kg; 60min; PAOLA-1 study) [32] for residual tumour (only)	6 cycles every 3 weeks, then for 15M	x	x									0.63 [7]	1,362 per 400 mg unit
**rOC (recurrent)** (median time until progression ~2 years [8], median OS ~30M [33])													0.73 [8]	
**Diagnostics** (CT-Scan)					x									Supplement Table S2
**Follow-up visits**					Every 3M clinical consultation & CT-Scan									Supplement Table S2

GYNAECOLOGIC	Medication	Application	10-years tunnel										Proportion	Costs [a] (EUR)
**rOC** *(continued)*			1	2	3	4	5	6	7	8	9	10		
**Chemotherapy & targeted therapy** [b]														
1st	Platin-based (e.g. carboplatin, 6 AUC mg/ml*Min; 15-60min)	6 cycles (every 4 weeks)			x									126 per 600 mg unit
	Anthracycline derivative (e.g. caelyx, 50mg/m^2; 60min)	6 cycles (every 3 weeks)			x									583 per 20 mg unit
	Anti-VEGF (bevacizumab, e.g. Avastin, 15mg/kg; 60min)	until progression, every 3 weeks (MITO16B-MaNGO; median PFS 11.8 M [34, 35]			x									1,362 per 400 mg unit
2nd	Platin-based (e.g. carboplatin, 6 AUC mg/ml*Min; 15-60min)	6 cycles (every 4 weeks)				x								126 per 600 mg unit
	Anthracycline derivative (e.g. caelyx, 50mg/m^2; 60min)	6 cycles (every 4 weeks)				x								583 per 20 mg unit
3rd	Platin-based (e.g. carboplatin, 4 AUC mg/ml*Min; 15-60min)	6 cycles, every 3 weeks				x								126 per 600 mg unit
	Gemcitabine (1,000mg/m^2; 30min)	6 cycles, every 3 weeks on day 1 and 8 of each 21-days cycle				x								195 per 2,000 mg unit
4th	Platin-based (e.g. carboplatin, 6 AUC mg/ml*Min; 15-60min)	6 cycles, every 4 weeks					x							126 per 600 mg unit

[a] Medication costs were obtained from the Swiss medication list, http://www.spezialitätenliste.ch [12]; sensitivity analysis of medication costs: ±20% (estimated variation)

Abbreviations: OC (ovarian cancer), rOC (recurrent ovarian cancer), M (months), CT (computer-tomography)

[b] Specifications for chemotherapy (cost) calculations: The amount of chemotherapy required for the cost estimate [mg] to reach a specific target [mg/m^2] was calculated using du Bois' formula for calculating body surface area [m^2] (body surface [m^2]=0.007184 x height [cm]^{0,725} x weight [kg]$^{0.425}$) [28] and assuming that the average Swiss woman is 166cm tall (https://www.conviva-plus.ch/?page=2046) and of normal weight (60kg) [9] resulting in a body surface area of 1.67m^2.

To achieve a target AUC of 6 for carboplatin chemotherapy, the following assumptions were made: The mean age of breast and ovarian cancer diagnosis for women with a *BRCA* mutation is 46 respectively 57 years [29], with a normal creatinine level in the blood serum of 1.1 mg/dL resulting in a glomerular filtration rate (GFR) of 60.5mL/min or 53.4mL/min, respectively, using the formula GFR[mL/min] = (((140-age[years])*weight[kg]) / (72*serum-creatinine[mg/dL]))*0.85(for women). Using the Calvert-formula (i.e. carboplatin-dose [mg] = TargetAUC [mg/ml*min] * (GFR [mL/min] + 25)) [30] resulted in a carboplatin dosage of 513mg for breast respectively 471mg for ovarian cancer, which was used for the cost estimation.

Results - Tables

Supplement Table S5 Summary of total BC and OC cost estimations in the model

**BC**	
BRCA1	**EUR**
BC in the 1. year	55,791
Annual costs of MBC [a]	57,607
BRCA2	
BC in the 1. year	47,645
Annual costs of MBC [a]	38,746
**OC**	
BRCA1/2	
OC in the 1. year (whereof surgery: about 21,000)	121,810
OC in the 2. year	82,163
Recurrent OC in the 1. year	60,037
Recurrent OC in the 2. year	34,891
Recurrent OC in the 3. year (excluding palliative care)	11,970

[a] Annual costs of MBC: overall total costs per year in the MBC state (overall median survival of MBC is approximately 3 years with a relative, age-adjusted 5-year survival rate of 27% (95% CI: 24-31%) [36])

Abbreviations: BC (breast cancer), MBC (metastatic breast cancer), OC (ovarian cancer)

Supplement Table S6 Results of sensitivity analysis for *BRCA1* and *BRCA2* concerning age of prophylactic measures

Strategy	Cost	QALY	LYs	dCost[a]	dQALYs[b]	dLYs[c]	ICER	dCost base case	dQALYs base case	dLYs base case
BRCA1: 35 years										
IS	138,684	15.86	20.01	reference			dominated	-2,608	1.38	1.39
CP	133,895	16.69	20.31	-4,790	0.83	0.29	dominated	-3,062	1.45	1.42
PBSO	116,715	17.97	21.42	-21,969	2.11	1.41	dominated	3,901	1.19	1.10
PBM	108,865	18.97	22.32	-29,819	3.11	2.31	dominated	-6,937	1.69	1.63
PBM & PBSO	76,345	20.67	24.29	-62,339	4.81	4.28	undominated	-294	1.43	1.34
BRCA1: 30 years										
IS	135,732	17.11	21.27	reference			dominated	-5,561	2.63	2.65
CP	130,639	18.00	21.57	-5,093	0.89	0.31	dominated	-6,318	2.76	2.69
PBSO	118,630	19.06	22.44	-17,102	1.96	1.18	dominated	5,817	2.28	2.12
PBM	102,898	20.45	23.75	-32,834	3.34	2.48	dominated	-12,904	3.17	3.06
PBM & PBSO	75,952	21.93	25.47	-59,780	4.82	4.20	undominated	-687	2.69	2.52
BRCA2: 35 years										
IS	97,624	17.07	21.40	reference			dominated	-4,621	1.56	1.56
PBSO	95,279	18.42	21.89	-2,345	1.35	0.49	dominated	-1,812	1.57	1.48
CP	75,455	19.13	22.92	-22,169	2.06	1.52	dominated	-3,024	1.55	1.48
PBM	67,916	20.75	24.28	-29,708	3.68	2.87	dominated	-2,647	1.51	1.42
PBM & PBSO	61,343	21.26	24.94	-36,281	4.19	3.54	undominated	573	1.41	1.31
BRCA2: 30 years										
IS	93,550	18.43	22.76	reference			dominated	-8,695	2.92	2.92
PBSO	93,537	19.78	23.18	-13	1.35	0.42	dominated	-3,554	2.94	2.77
CP	72,737	20.50	24.22	-20,814	2.07	1.45	dominated	-5,742	2.92	2.78
PBM	65,637	22.07	25.52	-27,913	3.64	2.75	dominated	-4,925	2.83	2.66
PBM & PBSO	61,817	22.50	26.09	-31,734	4.07	3.32	undominated	1,046	2.64	2.46

[a] dCosts: average difference in costs per women who opted for prophylactic measures compared to intensified surveillance (reference)
[b] dQALYs: average difference in QALYs per woman who opted for prophylactic measures compared to intensified surveillance (reference)
[c] dLYs: average difference in LYs per woman who opted for prophylactic measures compared to intensified surveillance (reference)
Abbreviations: QALYs (quality-adjusted life years), LYs (life years), ICER (incremental cost-effectiveness ratio), IS (intensified surveillance), PBM (prophylactic bilateral mastectomy), PBSO (prophylactic bilateral salpingo-oophorectomy), CP (chemoprevention)

Supplement Table S7 Results of sensitivity analysis for *BRCA1* and *BRCA2* concerning mortality rate of OC

v_TRates_OCtoDead_column								
**Values**	**Strategy**	**Costs**	**QALYs**	**dCosts[a]**	**dQALYs[b]**	**ICER**	**dCosts** base case	**dQALYs** base case
BRCA1								
Base case (age-specific) [c] - 30%	IS	141,702	14.55	reference		dominated	409	0.07
	CP	137,380	15.31	-4,322	0.76	dominated	423	0.07
	PBM	116,076	17.36	-25,626	2.81	dominated	274	0.08
	PBSO	112,929	16.81	-28,773	2.26	dominated	116	0.02
	PBM & PBSO	76,730	19.27	-64,972	4.71	undominated	91	0.02
BRCA2								
Base case (age-specific) [c] - 30%	IS	102,370	15.53	reference		dominated	125	0.02
	PBSO	97,120	16.85	-5,250	1.32	dominated	29	0.00
	CP	78,608	17.60	-23,762	2.07	dominated	130	0.02
	PBM	70,668	19.26	-31,702	3.73	dominated	105	0.02
	PBM & PBSO	60,801	19.86	-41,569	4.33	undominated	31	0.01

[a] dCosts: average difference in costs per women who opted for prophylactic measures compared to intensified surveillance (reference)
[b] dQALYs: average difference in QALYs per woman who opted for prophylactic measures compared to intensified surveillance (reference)
[c] values as specified in **Table 1**; estimation of 30% based on the SOLO1 study [31]
Abbreviations: QALYs (quality-adjusted life years), ICER (incremental cost-effectiveness ratio), IS (intensified surveillance), PBM (prophylactic bilateral mastectomy), PBSO (prophylactic bilateral salpingo-oophorectomy), CP (chemoprevention)

Supplement Table S8 Results of sensitivity analysis for *BRCA1* and *BRCA2* concerning costs of a reconstruction at a later time point

Strategy	Cost	QALYs	dCosts[a]	Dominance	dCosts base case
BRCA1 - later					
IS	141,995		reference	dominated	702
CP	137,591		-4,405	dominated	634
PBM	115,886		-26,109	dominated	84
PBSO	113,595		-28,400	dominated	781
PBM & PBSO	76,736		-65,260	undominated	96
BRCA2 - later					
IS	103,157		reference	dominated	912
PBSO	98,028		-5,129	dominated	937
CP	78,994		-24,162	dominated	516
PBM	70,686		-32,471	dominated	124
PBM & PBSO	60,900		-42,257	undominated	129

[a] dCosts: average difference in costs per women who opted for prophylactic measures compared to intensified surveillance (reference)

Abbreviations: QALYs (quality-adjusted life years), ICER (incremental cost-effectiveness ratio), IS (intensified surveillance), PBM (prophylactic bilateral mastectomy), PBSO (prophylactic bilateral salpingo-oophorectomy), CP (chemoprevention)

Supplement Table S9 Overview of results (QALYs and LYs) that were found in modelling studies

	QALYs	LYs	QALYs	LYs
Note: data presented in comparison to surveillance and bold represents dominant strategy				
**Study / Risk-reduction uptake**	**Grann [37]**	**40 years**		
Population	*BRCA1/BRCA2*			
CP	2.2	0.7		
PBSO	3.1	1.4		
PBM	1.0	1.4		
PBM & PBSO	0.8	2.3		
**Study / Risk-reduction uptake**	**Anderson [38]**	**35 years**		
Population	*BRCA1*		*BRCA2*	
CP	1.1	0.5	0.9	0.7
**PBSO**	**2.8**	1.8	**1.3**	1.3
PBM	0.8	1.0	1.1	1.4
**PBM & PBSO**	1.8	**2.6**	1.9	**2.2**
	**Sensitivity analysis: risk-reduction uptake at age 40 years**			
**PBSO**	**dominated**		**dominated**	
**PBM & PBSO**		**dominated**		**dominated**
**Risk-reduction uptake / Study**	**Norum [2]**	**35 years**		
Population	*BRCA1/BRCA2*			
PBSO		3.1		
PBM & PBSO		6.4		
**Study / Risk-reduction uptake**	**Muller [3]**	**30 years**		
Population	*BRCA1/BRCA2*			
PBSO	1.7	1.6		
PBM	1.2	0.8		
**PBM & PBSO**	**2.7**	**2.2**		
**Study / Risk-reduction uptake**	**present study**	**40 years**		
Population	*BRCA1*		*BRCA2*	
CP	0.8	0.3	2.1	1.6
PBSO	2.3	1.7	1.3	0.6
PBM	2.8	2.1	3.7	3.0
**PBM & PBSO**	**4.8**	**4.3**	**4.3**	**3.8**

Abbreviations: QALYs (quality-adjusted life years), LYs (life years), PBM (prophylactic bilateral mastectomy), PBSO (prophylactic bilateral salpingo-oophorectomy), CP (chemoprevention)

Results - Figures

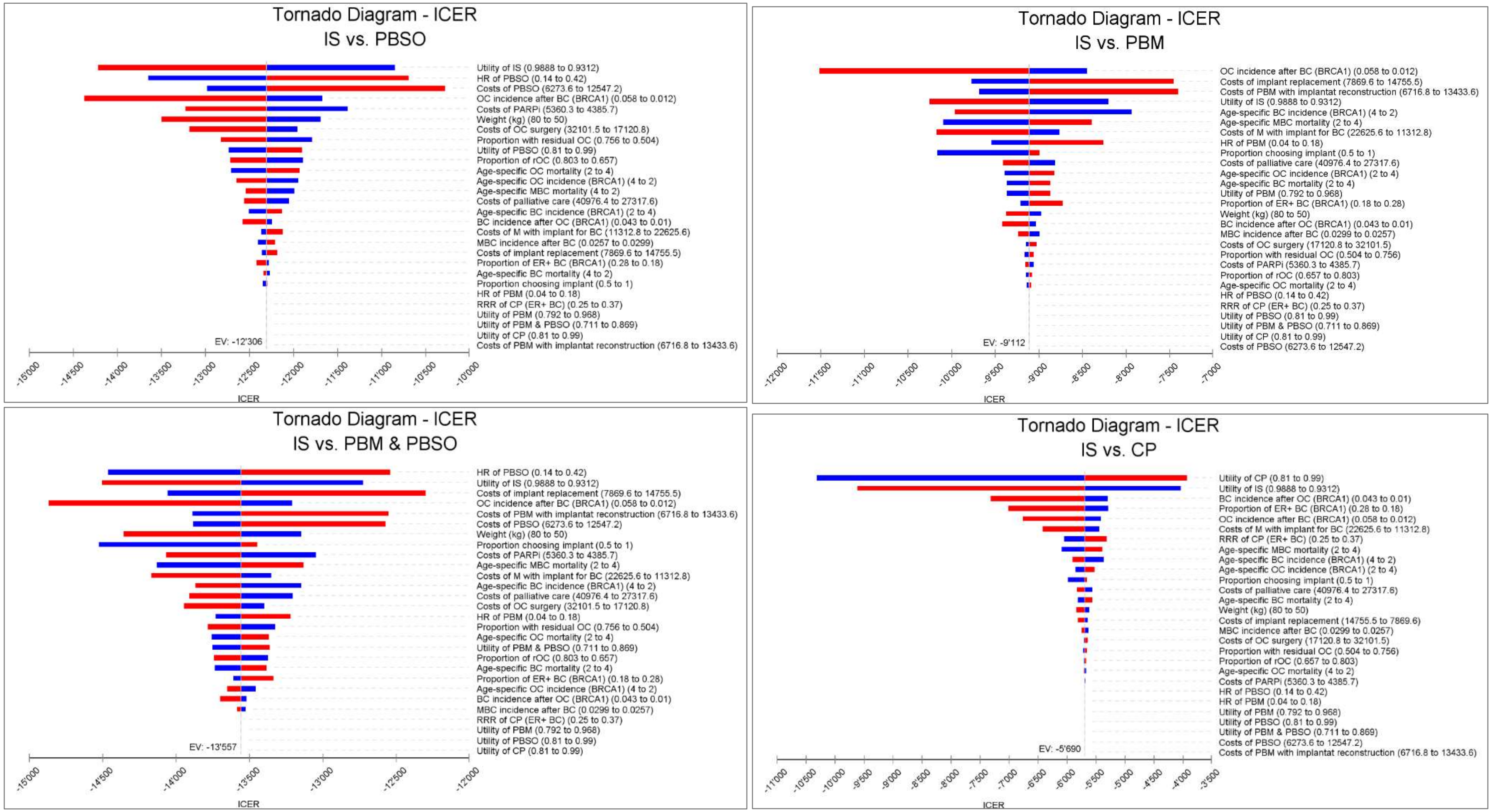

Supplement Figure S1 Tornado diagrams showing parameters (excluding age) and their influence on the outcome of the cost-effectiveness for the different prophylactic strategies in comparison to the reference group in *BRCA1* mutation carriers (red: higher than base case, blue: lower than base case). Abbreviations: IS (intensified surveillance), PBM (prophylactic bilateral mastectomy), PBSO (prophylactic bilateral salpingo-oophorectomy), CP (chemoprevention), ICER (incremental cost-effectiveness ratio), OC (ovarian cancer), rOC (recurrent OC), BC (breast cancer), MBC (metastatic breast cancer), M (mastectomy), HR (hazard ration), RRR (relative risk reduction), ER+ (estrogen receptor positive), PARPi (Poly-ADP-Ribose-Polymerase Inhibitor), EV (expected value).

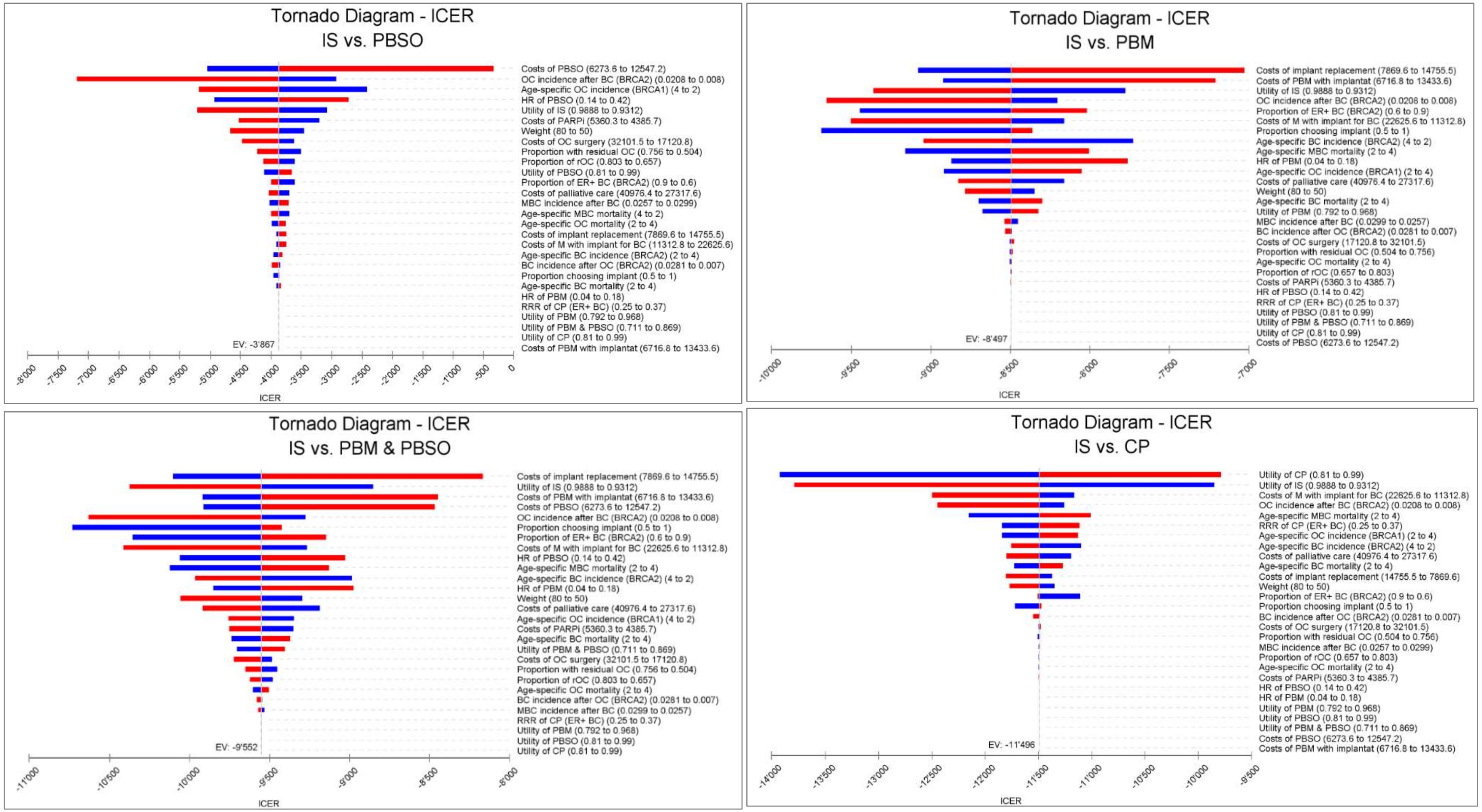

Supplement Figure S2 Tornado diagrams showing parameters (excluding age) and their influence on the outcome of the cost-effectiveness for the different prophylactic strategies in comparison to the reference group in *BRCA2* mutation carriers (red: higher than base case, blue: lower than base case). Abbreviations: IS (intensified surveillance), PBM (prophylactic bilateral mastectomy), PBSO (prophylactic bilateral salpingo-oophorectomy), CP (chemoprevention), ICER (incremental cost-effectiveness ratio), OC (ovarian cancer), rOC (recurrent OC), BC (breast cancer), MBC (metastatic breast cancer), M (mastectomy), HR (hazard ration), RRR (relative risk reduction), ER+ (estrogen receptor positive), PARPi (Poly-ADP-Ribose-Polymerase Inhibitor), EV (expected value).

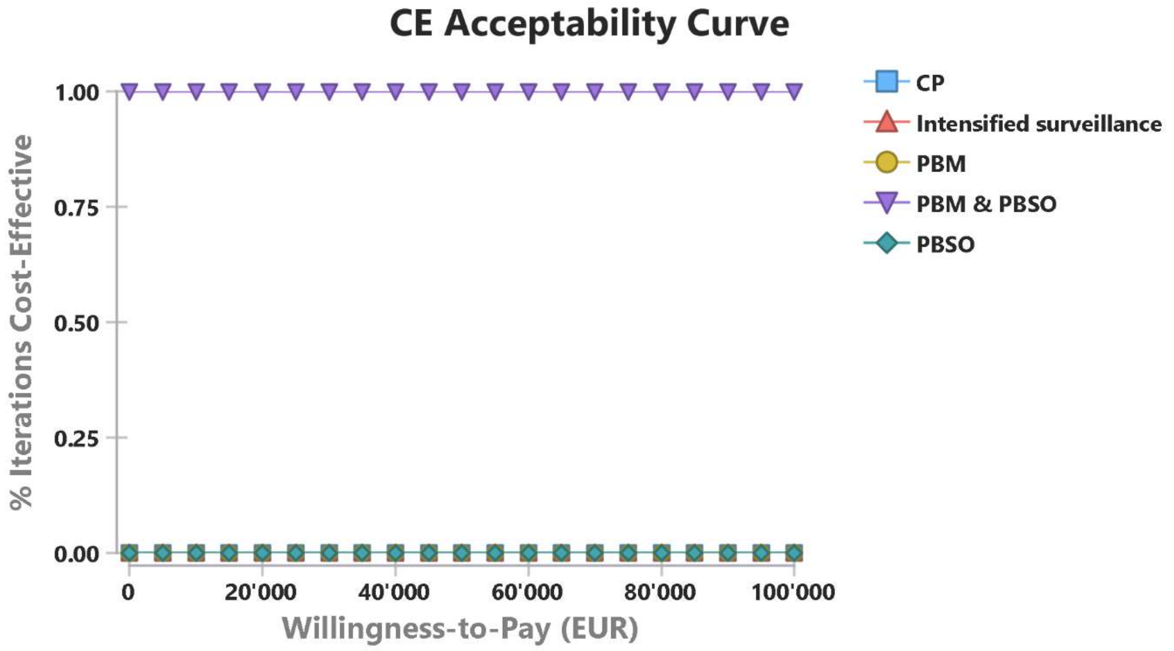

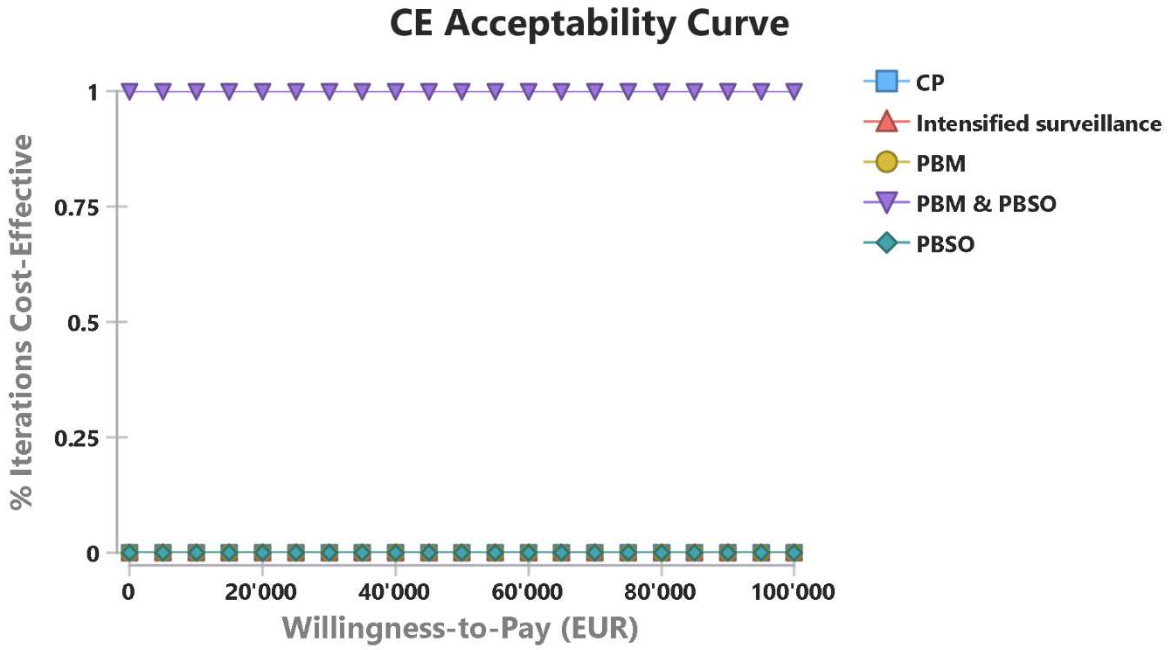

Supplement Figure S3 Cost-effectiveness acceptability curve (CEAC) for *BRCA1* (top) and *BRCA2* (bottom). Abbreviations: CP (chemoprevention), PBM (prophylactic bilateral mastectomy), PBSO (prophylactic bilateral salpingo-oophorectomy). Curves for all strategies except PBM & PBSO are overlaid.

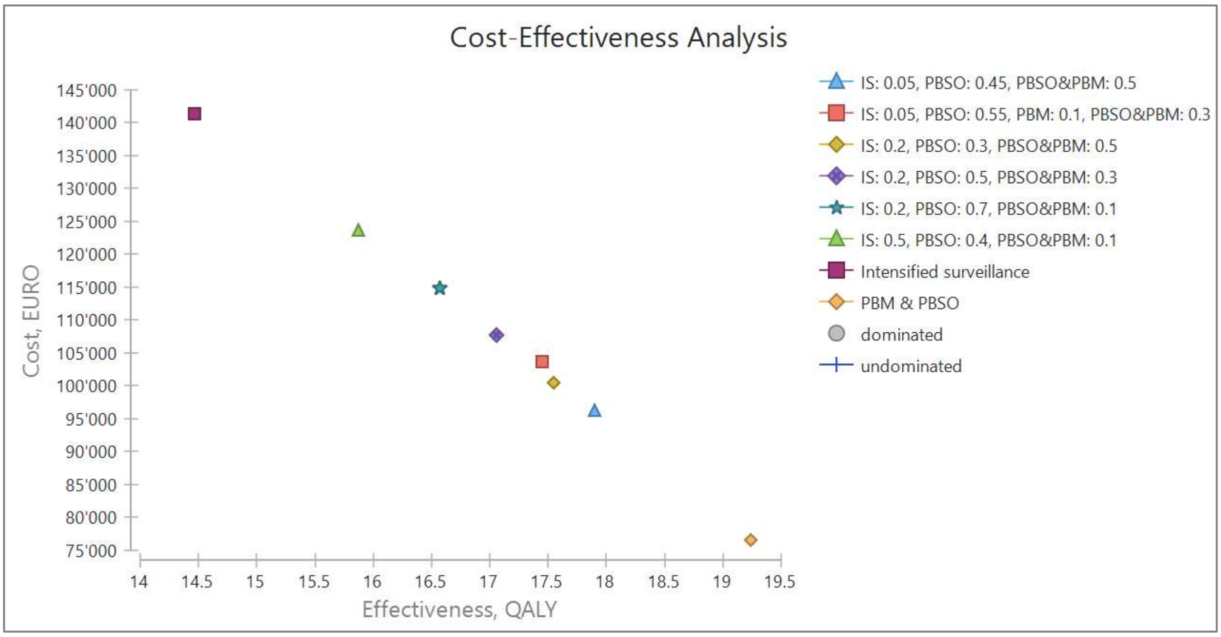

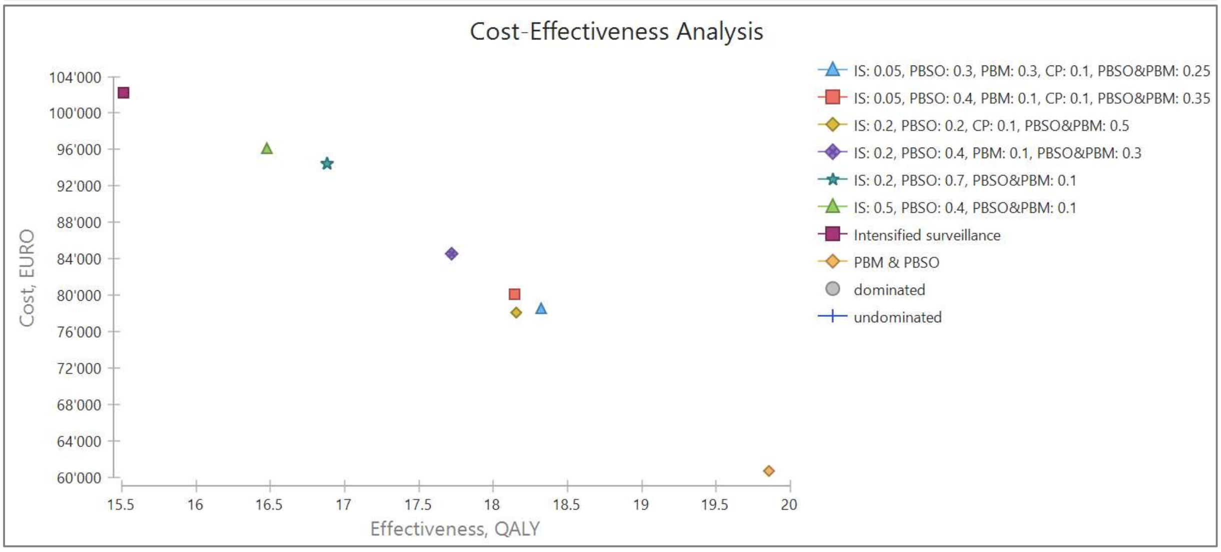

Supplement Figure S4 Results of scenario analysis showing different combinations of risk-reducing strategies for *BRCA1* (top) and *BRCA2* (bottom) together with the reference group (IS) and undominated strategy (PBSO & PBM). Abbreviations: QALYs (quality-adjusted life years), IS (intensified surveillance), CP (chemoprevention), PBM (prophylactic bilateral mastectomy), PBSO (prophylactic bilateral salpingo-oophorectomy).

References

1. Grann, V.R., et al., *Comparative effectiveness of screening and prevention strategies among BRCA1/2-affected mutation carriers.* Breast Cancer Res Treat, 2011. **125**(3): p. 837-47.
2. Norum, J., et al., *Prophylactic bilateral salpingo-oophorectomy (PBSO) with or without prophylactic bilateral mastectomy (PBM) or no intervention in BRCA1 mutation carriers: a cost-effectiveness analysis.* Eur J Cancer, 2008. **44**(7): p. 963-71.
3. Muller, D., et al., *Cost-effectiveness of different strategies to prevent breast and ovarian cancer in German women with a BRCA 1 or 2 mutation.* Eur J Health Econ, 2018. **19**(3): p. 341-353.
4. Yao, K., et al., *Nipple-sparing mastectomy in BRCA1/2 mutation carriers: an interim analysis and review of the literature.* Ann Surg Oncol, 2015. **22**(2): p. 370-6.
5. Kuchenbaecker, K.B., et al., *Associations of common breast cancer susceptibility alleles with risk of breast cancer subtypes in BRCA1 and BRCA2 mutation carriers.* Breast Cancer Res, 2014. **16**(6): p. 3416.
6. Spurdle, A.B., et al., *Refined histopathological predictors of BRCA1 and BRCA2 mutation status: a large-scale analysis of breast cancer characteristics from the BCAC, CIMBA, and ENIGMA consortia.* Breast Cancer Res, 2014. **16**(6): p. 3419.
7. Klar, M., et al., *Prognostic factors in young ovarian cancer patients: An analysis of four prospective phase III intergroup trials of the AGO Study Group, GINECO and NSGO.* Eur J Cancer, 2016. **66**: p. 114-24.
8. Landrum, L.M., et al., *Prognostic factors for stage III epithelial ovarian cancer treated with intraperitoneal chemotherapy: a Gynecologic Oncology Group study.* Gynecol Oncol, 2013. **130**(1): p. 12-8.
9. *Bundesamt für Statistik (BFS) - Body Mass Index (BMI) nach Geschlecht, Alter, Bildungsniveau, Sprachgebiet* 30.10.2018; Available from: https://www.bfs.admin.ch/bfs/de/home/statistiken/gesundheit/determinanten/uebergewicht.assetdetail.6466017.html.
10. *Swiss DRG catalogue*. Available from: https://www.swissdrg.org/de.
11. *Swiss Tarmed catalogue*. Available from: https://www.tarmed-browser.ch/de/leistungen.
12. *Bundesamt für Gesundheit (BAG) - Swiss medication list (Spezialitätenliste; SL)*. Available from: http://www.spezialitätenliste.ch.
13. Turner, N.C., et al., *Overall Survival with Palbociclib and Fulvestrant in Advanced Breast Cancer.* N Engl J Med, 2018. **379**(20): p. 1926-1936.
14. Finn, R.S., et al., *The cyclin-dependent kinase 4/6 inhibitor palbociclib in combination with letrozole versus letrozole alone as first-line treatment of oestrogen receptor-positive, HER2-negative, advanced breast cancer (PALOMA-1/TRIO-18): a randomised phase 2 study.* Lancet Oncol, 2015. **16**(1): p. 25-35.
15. Cristofanilli, M., et al., *Fulvestrant plus palbociclib versus fulvestrant plus placebo for treatment of hormone-receptor-positive, HER2-negative metastatic breast cancer that progressed on previous endocrine therapy (PALOMA-3): final analysis of the multicentre, double-blind, phase 3 randomised controlled trial.* Lancet Oncol, 2016. **17**(4): p. 425-439.
16. Yardley, D.A., et al., *Everolimus plus exemestane in postmenopausal patients with HR(+) breast cancer: BOLERO-2 final progression-free survival analysis.* Adv Ther, 2013. **30**(10): p. 870-84.
17. Robson, M.E., et al., *OlympiAD final overall survival and tolerability results: Olaparib versus chemotherapy treatment of physician's choice in patients with a germline BRCA mutation and HER2-negative metastatic breast cancer.* Ann Oncol, 2019. **30**(4): p. 558-566.
18. Schmid, P., et al., *Atezolizumab and Nab-Paclitaxel in Advanced Triple-Negative Breast Cancer.* N Engl J Med, 2018. **379**(22): p. 2108-2121.
19. Robson, M., et al., *Olaparib for Metastatic Breast Cancer in Patients with a Germline BRCA Mutation.* N Engl J Med, 2017. **377**(6): p. 523-533.
20. Swain, S.M., et al., *Pertuzumab, trastuzumab, and docetaxel in HER2-positive metastatic breast cancer.* N Engl J Med, 2015. **372**(8): p. 724-34.
21. Perez, E.A., et al., *Trastuzumab emtansine with or without pertuzumab versus trastuzumab with taxane for human epidermal growth factor receptor 2-positive advanced breast cancer: Final results from MARIANNE.* Cancer, 2019. **125**(22): p. 3974-3984.
22. Baselga, J., et al., *Pertuzumab plus trastuzumab plus docetaxel for metastatic breast cancer.* N Engl J Med, 2012. **366**(2): p. 109-19.
23. Verma, S., et al., *Trastuzumab emtansine for HER2-positive advanced breast cancer.* N Engl J Med, 2012. **367**(19): p. 1783-91.
24. Jackisch, C., et al., *Impact of trastuzumab treatment beyond disease progression for advanced/metastatic breast cancer on survival - results from a prospective, observational study in Germany.* Breast, 2014. **23**(5): p. 603-8.

25. Cameron, D., et al., *A phase III randomized comparison of lapatinib plus capecitabine versus capecitabine alone in women with advanced breast cancer that has progressed on trastuzumab: updated efficacy and biomarker analyses.* Breast Cancer Res Treat, 2008. **112**(3): p. 533-43.
26. von Minckwitz, G., et al., *Trastuzumab beyond progression: overall survival analysis of the GBG 26/BIG 3-05 phase III study in HER2-positive breast cancer.* Eur J Cancer, 2011. **47**(15): p. 2273-81.
27. Larionov, A.A., *Current Therapies for Human Epidermal Growth Factor Receptor 2-Positive Metastatic Breast Cancer Patients.* Front Oncol, 2018. **8**: p. 89.
28. Du Bois, D. and E.F. Du Bois, *Clinical calorimetry: tenth paper a formula to estimate the approximate surface area if height and weight be known.* Archives of internal medicine, 1916. **17**(6_2): p. 863-871.
29. Kuchenbaecker, K.B., et al., *Risks of Breast, Ovarian, and Contralateral Breast Cancer for BRCA1 and BRCA2 Mutation Carriers.* JAMA, 2017. **317**(23): p. 2402-2416.
30. Calvert, A.H., et al., *Carboplatin dosage: prospective evaluation of a simple formula based on renal function.* J Clin Oncol, 1989. **7**(11): p. 1748-56.
31. Moore, K., et al., *Maintenance Olaparib in Patients with Newly Diagnosed Advanced Ovarian Cancer.* N Engl J Med, 2018. **379**(26): p. 2495-2505.
32. Ray-Coquard, I., et al., *Olaparib plus Bevacizumab as First-Line Maintenance in Ovarian Cancer.* N Engl J Med, 2019. **381**(25): p. 2416-2428.
33. Ledermann, J.A., et al., *Overall survival in patients with platinum-sensitive recurrent serous ovarian cancer receiving olaparib maintenance monotherapy: an updated analysis from a randomised, placebo-controlled, double-blind, phase 2 trial.* Lancet Oncol, 2016. **17**(11): p. 1579-1589.
34. Pignata, S., et al., *Chemotherapy plus or minus bevacizumab for platinum-sensitive ovarian cancer patients recurring after a bevacizumab containing first line treatment: the randomized phase 3 trial MITO16B-MaNGO OV2B-ENGOT OV17.* J Clin Oncol, 2018. **36**(suppl): p. 5506.
35. Daniele, G., et al., *Feasibility and outcome of interval debulking surgery (IDS) after carboplatin-paclitaxel-bevacizumab (CPB): A subgroup analysis of the MITO-16A-MaNGO OV2A phase 4 trial.* Gynecol Oncol, 2017. **144**(2): p. 256-259.
36. Bouchardy Magnin, C., M. Lorez, and V. Arndt, *Effects of age and stage on breast cancer survival in Switzerland.* Bulletin suisse du cancer, 2015(2): p. 152-157.
37. Grann, V.R., et al., *Effect of prevention strategies on survival and quality-adjusted survival of women with BRCA1/2 mutations: an updated decision analysis.* J Clin Oncol, 2002. **20**(10): p. 2520-9.
38. Anderson, K., et al., *Cost-effectiveness of preventive strategies for women with a BRCA1 or a BRCA2 mutation.* Ann Intern Med, 2006. **144**(6): p. 397-406.
